# Effect of Strength Training on Oxidative Stress and the Correlation of the Same with Forearm Vasodilatation and Blood Pressure of Hypertensive Elderly Women: A Randomized Clinical Trial

**DOI:** 10.1371/journal.pone.0161178

**Published:** 2016-08-16

**Authors:** Filipe Fernandes Oliveira Dantas, Maria do Socorro Brasileiro-Santos, Rafael Marinho Falcão Batista, Leone Severino do Nascimento, Lúcio Roberto Cançado Castellano, Raphael Mendes Ritti-Dias, Kenio Costa Lima, Amilton da Cruz Santos

**Affiliations:** 1 Graduate Associate Program in Physical Education–Universidade Federal da Paraíba, João Pessoa, Brazil; 2 Research Laboratory for Physical Training Applied to Performance and Health–Universidade Federal da Paraíba, João Pessoa, Brazil; 3 Human Immunology Research and Education Group, Health Technical School–Universidade Federal da Paraíba, João Pessoa, Brazil; 4 Hospital Israelita Albert Einstein, São Paulo, Brazil; 5 Postgraduate Program in Public Health–Universidade Federal do Rio Grande do Norte, Natal, Brazil; Tokyo Institute of Technology, JAPAN

## Abstract

The aim of the study was to evaluate the effect of strength training on oxidative stress and the correlation of the same with forearm vasodilatation and mean blood pressure of hypertensive elderly women, at rest (basal) and during a static handgrip exercise. Insufficiently active hypertensive elderly women (N = 25; mean age = 66.1 years) were randomized into a 10 week strength training group (n = 13) or control (n = 12) group. Plasma malondialdehyde (MDA), total antioxidant capacity (TAC), plasma nitrite (NO_2_^-^), forearm blood flow (FBF), mean blood pressure (MBP) and vascular conductance ([FBF / MBP] x 100) were evaluated before and after the completion of the interventions. The strength training group increased the TAC (pre: Median = 39.0; Interquartile range = 34.0–41.5% vs post: Median = 44.0; Interquartile range = 38.0–51.5%; *p* = 0.006) and reduced the MDA (pre: 4.94 ± 1.10 μM vs post: 3.90 ± 1.35 μM; *p* = 0.025; CI-95%: -1.92 –-0.16 μM). The strength training group increased basal vascular conductance (VC) (pre: 3.56 ±0.88 units vs post: 5.21 ±1.28 units; *p* = 0.001; CI-95%: 0.93–2.38 units) and decreased basal MBP (pre: 93.1 ±6.3 mmHg vs post: 88.9 ±5.4 mmHg; *p* = 0.035; CI-95%: -8.0 –-0.4 mmHg). Such changes were also observed during static handgrip exercise. A moderate correlation was observed between changes in basal VC and MBP with changes in NO_2_^-^ (ΔVC → r = -0.56, *p* = 0.047; ΔMBP → r = -0.41, *p* = 0.168) and MDA (ΔVC → r = 0.64, *p* = 0.019; ΔMBP → r = 0.31, *p* = 0.305). The strength training program reduced the oxidative stress of the hypertensive elderly women and this reduction was moderately correlated with their cardiovascular benefits.

***Trial Registration*:** ensaiosclinicos.gov.br RBR-48c29w

## Introduction

Aging alters vascular structure and function [1, 2], changes that are potentiated by hypertension [3]. Hypertensive elderly individuals, besides having elevated blood pressure, both when at rest and during exercise [4], also display reduced peripheral vasodilatation [5].

Elevated blood pressure (BP) in elderly individuals can be responsible for end organ damage, including chronic kidney disease, retinopathy, cerebrovascular disease, cardiac dysfunction and atrial fibrillation [6]. Furthermore, increased BP response during exercise is associated with cardiovascular and cerebrovascular events, which can occur during or after exercise [7]. Reduced peripheral vasodilatation in elderly individuals results in a decrease in the oxygen supply to skeletal muscle [8], which may be responsible for the increased fatigue and reduced mobility of these individuals [9].

Problems related to elevated BP can be more pronounced in hypertensive elderly women (HEW), among whom BP is less easy to control [10]. In addition, due to the loss of the protective effect of endogenous estrogen, which preserves nitric oxide bioavailability, HEW may present greater impairment in peripheral vasodilatation [11].

It has been recommended that strength training (ST) is included in a global exercise program for older individuals [12], based on its effects on muscle function [13–15]. However, few randomized controlled trials have been conducted investigating the chronic effects of ST on the cardiovascular variables of the HEW. Among these few studies, we highlight the study of Moreira, Cucato [16] and Mota, de Oliveira [17], which showed that ST programs can chronically reduce the BP of HEW. In addition, Collier, Frechette [18] showed that moderate intensity ST (3 days/week for 4 weeks) may be a more effect treatment option for hypertension among elderly women because it achieves greater reductions in diastolic BP and significant increases in resting forearm blood flow, without concomitant increases in arterial stiffness, compared with their male counterparts. However, the relationship between the cardiovascular changes promoted by ST and the oxidative stress of HEW has been little explored.

The literature shows that hypertensive elderly individuals present increased oxidative stress [19, 20], one possible mechanism by which the prevalence of hypertension is increased and peripheral vasodilatation is impaired during aging [21]. ST has been suggested as a mode of exercise in which chronic practice has displayed a protective effect against oxidative stress among the elderly [22].

However, whether the protective effect promoted by ST is correlated to cardiovascular benefits among HEW is still unknown. In this way, the present study tested the hypothesis that the reduction in oxidative stress is correlated to cardiovascular benefits in HEW who practice ST. Therefore, the aim of the present study was to evaluate the effect of ST on oxidative stress and the correlation of the same with the forearm vasodilatation and mean blood pressure (MBP) of the HEW, both at rest (basal) and during a static handgrip exercise.

## Materials and Methods

### Participants

Insufficiently active HEW were recruited between July 2014 and January 2015 through the Federal University of Paraiba, Brazil, and through advertisements in the media. The sample size was calculated based on Independent Samples T–test, using a moderate effect size (0.50). We considered plasma malondialdehyde (MDA) concentration (one of the primary end points) to adopt this effect size, which represents that someone from Control group with an average score, would have a higher score than 69% of the people from ST group [23]. This represents a higher risk to mortality [24]. Furthermore, was assumed a power of 80% (for a one-tailed alpha value = 0.05). Additionally, it was adopted a moderately strong correlation among the repeated measures (r = 0.50), considering that the post-intervention MDA mean values is explained about 25% by pre-intervention values. According these informations, the power analysis yielded a sample size of 21 participants. Our sample included 25 participants that were randomized into either a 10-week ST group or a non-exercise control group.

Non-smoking females aged between 60 and 75 years of age, who were insufficiently active for at least 6 months (<2 days per week of structured physical activity, no regular ongoing resistance exercise practice) and hypertensive using drug treatment were included. Participants were excluded if they presented hematologic disease, peripheral vascular disease, stroke, fasting plasma glucose ≥ 126 mg/dL, consumed more than 60 g of alcohol (corresponding to half a liter of wine) per day, or were undergoing estrogen replacement therapy.

### Randomization

After giving consent, eligible and not excluded HEW were randomized into a ST or non-exercise control group. Simple randomization was used to allocate the HEW into the study groups. Each elderly woman had access to a file containing identically colored envelopes containing information about the group they would join. The HEW who were randomly assigned to the control group maintained their usual habits throughout the study. For ethical reasons, the control group was, at the end of the study, invited to participate in the ST program applied in the other group.

### Procedures

The general characteristics data and all pre-intervention assessments were conducted prior to randomizing the participants into the ST group or control group. Both assessors and participants were blinded regarding group placement at the time of the baseline assessments.

The general characteristics data was collected in the first meeting with the participants. Subsequently, the volunteers were informed about the necessity of fasting for at least 12 hours so that blood could be collected the next day for laboratory tests. Once the results of the blood tests had been obtained, the exclusion criteria were applied. A date was scheduled for the HEW who were not excluded to assess forearm vasodilatation by venous occlusion plethysmography. Before collection of forearm vasodilatation data, the HEW were advised not to perform any kind of exercise on the day before the procedure. It was also recommended that they maintained their usual alimentary habits, and avoided ingesting stimulant drinks, such as coffee, chocolate, soda or alcohol. All assessments were completed by all participants in the pre and post intervention period, and the assessors were blinded with respect to the groups of the HEW.

### Training intervention

The 10-week ST program was led by physical education professionals with experience in the field of ST. The supervised group sessions were held twice a week for the first five weeks, and after this initial period the frequency increased to three times per week. The techniques of the exercises were taught to participants in two training sessions separated by an interval of 48 hours, carried out prior to the beginning of the training program. These sessions were intended to make the participants familiar with the exercises. In the familiarization sessions, ten repetitions of each exercise were performed, with the lowest possible load on each device, teaching the elderly women to adopt the correct body positioning, range of motion (considering the individual limitations), as well as employing a proper breathing pattern while performing exercises (avoiding the Valsalva maneuver, breathing in the relaxation phase and exhaling in the contraction phase). Additionally, these sessions had the purpose of teaching how to correctly interpret the perceived exertion scale (OMNI-RES), adapted for ST [25], so that the intensity of training of individuals could be properly monitored (5–7 OMNI-RES scale). To facilitate the understanding of this subjective scale, the 1RM (maximal repetition) test was applied to the Bench Press and Seated Leg Press exercises, according to protocols previously described elsewhere [26].

The ST program consisted of nine exercises, which were carried out in the following order: Seated Leg Press; Seated Rowing Machine; Trunk Flexion; Knee Flexion Machine, Bench Press, Trunk Extension Machine, Push Press, Standing Plantar Flexion and Front Pulldown. The overall planning of variables relating to the ST protocol used in this study are included in S1 Table.

### End points

The primary end points of this trial were oxidative stress, forearm vasodilatation and MBP. Another secondary end point was nitric oxide bioavailability.

### Measurements

#### Evaluation of oxidative stress

Evaluation of oxidative stress was conducted by analyzing plasma malondialdehyde (MDA) and total antioxidant capacity (TAC). The oxidant activity of MDA was quantified by the thiobarbituric acid reaction with the decomposition products of hydroperoxide. This technique has coefficient of variation (CV) measurement error is following: intra-trial CV = 8.1%; inter-trial CV = 8.2%. For more details see reference [27]. The TAC results were described as the percentage of antioxidant activity (%TAC). This technique has CV measurement error is following: intra-trial CV = 3.0%; inter-trial CV = 5.1%. For details of procedures see reference [28].

#### Forearm vasodilatation and mean blood pressure

For the evaluation of the forearm vasodilatation the HEW were initially submitted to the maximal voluntary contraction test (MVC) using a handgrip. This test was used to determine the load of the static handgrip exercise (described later). After application of this test, the elderly individuals were placed in the supine position on a stretcher so that the equipment used to assess forearm vasodilatation could be installed. This was measured by venous occlusion plethysmography, which can identify forearm blood flow (FBF) through forearm vasodilatation. The methodological procedures of venous occlusion plethysmography have been described elsewhere [29]. Additionally, after placement of the plethysmography instruments, a pressure cuff (DIXTAL Medical^®^) was placed on the lower third of the leg of the HEW. The function of this cuff was to record BP (systolic, diastolic and mean) each minute during FBF analysis, which also allowed vascular conductance (VC) ([forearm blood flow / mean blood pressure] x 100) data to be collected.

Following the MVC test and instrumentation procedures, each HEW remained lying on the stretcher for 10 minutes until she returned to a normal resting condition. After this period, FBF and BP recording began. Such records were carried out as follows:

1. Basal: During initial instrumentation, as well as the placement of the plethysmography materials on the non-dominant arm, surface electrodes were placed on the chest (bipolar positions–derivation D2) to capture electrocardiographic signals. Electrocardiogram (ECG), FBF and BP data was recorded for three minutes. Both FBF and BP were analyzed as the mean of the three minute measure.
2. Static handgrip exercise (SHE): After basal measurements, SHE of the dominant arm was performed for three minutes, at an intensity of 30% MVC. This maneuver has the ability to increase muscle sympathetic nerve activity (MSNA), which is especially mediated by central command and the exercise pressor reflex (muscle mechanoreceptors and metaboreceptors) [30]. Both FBF and BP were analyzed each minute during exercise.

#### Evaluation of nitric oxide bioavailability

Nitric oxide bioavailability was evaluated by measurement of plasma nitrite (NO_2_^-^) which was performed by a spectrophotometric procedure based on the Griess reaction. The Griess Reagent System is based on a chemical reaction involving sulfanilamide and N-1-napthylethylenediamine dihydrochloride (NED) under acidic (phosphoric acid) conditions. This technique has CV measurement error is following: intra-trial CV = 6.3%; inter-trial CV = 5.7%. For more details of this procedure, see S1 File.

#### Analysis of biochemical parameters

The glucose, triglycerides, total cholesterol and HDL—cholesterol values were measured with LabMax 240^®^ equipment, following the standard operating procedure established for the biochemical evaluation of each measure and the proper use of reagents. The LDL—cholesterol value was calculated by the Friedewald formula (LDL-C = total cholesterol—HDL-C—[Triglycerides / 5]). For each blood parameter the CV measurement error is following: glucose (intra-trial CV = 0.62%; inter-trial CV = 1.66%), total cholesterol (intra-trial CV = 1.20%; inter-trial CV = 2.10%), HDL–cholesterol (intra-trial CV = 0.40%; inter-trial CV = 1.10%), triglycerides (intra-trial CV = 1.04%; inter-trial CV = 1.60%).

### Bioethics

The Ethics Research Committee of the Health Science Center of the Federal University of Paraiba, Brazil (Protocol 0216/14 –CAAE: 30878514.0.0000.5188) approved the study protocol. All participants were informed about the aims, procedures, benefits and potential risks of the trial, and agreed to participate and signed an informed consent form. In addition, the study was registered at www.ensaiosclinicos.gov.br (RBR-48c29w).

### Data analysis

Continuous variables are presented as mean values ± standard deviation (SD) (normal distribution) or median and interquartile range (non-normal distribution), while categorical variables are presented as frequencies. Data was analyzed using SPSS 21.0 software using an intent-to-treat approach. The verification of data normality was assessed by the Shapiro-Wilk test.

Continuous variables with normal distribution were evaluated by Split-Plot ANOVA (SPANOVA) mixed design. If significant interactions were found (group*time), post hoc tests were applied between the comparison pairs, through the Student T test for dependent samples (intragroup) and the Student T test for independent samples (intergroup). When the analysis in question involved multiple comparisons, the *p* values were submitted to Bonferroni correction. For these variables, the effect size (ES) was calculated in order to assess the magnitude of the difference between groups (training vs control), in the post-intervention period, according to criteria adopted by Sullivan and Feinn [23]. Additionally, for these variables the correlation was analyzed by Pearson's correlation coefficient (r). For the significant correlations the predictive power of the independent variable on the dependent variable was analyzed by simple linear regression analysis.

For the continuous variables without normal distribution, the evaluation was conducted by the Wilcoxon (intragroup), and U Mann-Whitney (intergroup) tests. In this case the correlation was analyzed by Spearman correlation coefficient (rho). Fisher's exact test was used to compare the proportion of use of the different types of medication between the groups. In all situations, a significance value less than 5% was considered.

## Results

The HEW of the ST group and non-exercise control group were, respectively, 64.7 ± 4.7 and 67.7 ± 5.6 years old (*p* = 0.161). Table 1 shows their baseline characteristics.

**Table 1:** General characteristics of hypertensive elderly women.

Variables	ST group (n = 13)		Control group (n = 12)		*p–value* (Interaction)
	Pre	Post	Pre	Post	
Body mass index (Kg/m^2^)	28.6 ± 3.2	28.4 ± 3.0	27.7 ± 3.7	27.6 ± 3.8	0.550
Bench Press exercise (1RM load–Kg)	23.1 ± 4.8	32.5 ± 5.9 *‡	22.3 ± 4.8	21.7 ± 4.3	**<0.001**
Seated Leg Press exercise (1RM load–Kg)	57.5 ± 25.7	83.7 ± 25.1 *‡	62.0 ± 13.9	60.4 ± 13.4	**<0.001**
Systolic blood pressure (mmHg)	142.9 ± 13.1	137.1 ± 12.2	139.9 ± 10.3	144.9 ± 14.1	0.050
Diastolic blood pressure (mmHg)	68.2 ± 6.2	64.9 ± 5.1 *‡	67.4 ± 9.5	72.0 ± 7.7	**0.016**
**Biochemical parameters**					
Glucose (mg/dL)	100.5 ± 13.7	97.8 ± 11.7	86.7 ± 11.0	89.2 ± 14.2	0.257
Total cholesterol (mg/dL)	218.2 ± 32.3	215.5 ± 40.1	225.2 ± 50.9	213.3 ± 47.5	0.646
HDL–Cholesterol (mg/dL)	46.8 ± 8.1	55.0 ± 7.6	45.6 ± 12.1	46.4 ± 9.6	0.096
LDL–Cholesterol (mg/dL)	138.2 ± 29.2	127.8 ± 37.4	148.3 ± 47.4	138.8 ± 38.9	0.963
Triglycerides (mg/dL)	166.7 ± 61.6	162.5 ± 73.2	157.7 ± 44.9	141.4 ± 42.1	0.459
**Drugs**					
**Use of only one drug (n, %)**					
Beta blockers (n,%)	2 (8.0)		3 (12.0)		
Alpha-2 agonist (n,%)	1 (4.0)		0 (0)		
**Antagonists of angiotensin II (n,%)**	3 (12.0)		3 (12.0)		
ACE inhibitors (n,%)	1 (4.0)		0 (0)		
**Combined use of two drugs (n,%)**					
Thiazide diuretics + Antagonists of angiotensin II (n,%)	2 (8.0)		3 (12.0)		
Thiazide diuretics + ACE inhibitors (n,%)	2 (8.0)		3 (12.0)		
Antagonists of angiotensin II + Calcium channel antagonists (n,%)	2 (8.0)		0 (0)		

*Note*: ACE: Angiotensin Converting Enzyme. Values are mean ± SD or percentages. Bold values represent significant interaction.

* *p* < 0.05 between periods within group

‡ p < 0.05 between groups within period.

The groups were similar in the pre-intervention period for most of the variables. In relation to Bench Press exercise, a significant interaction (group*time) was observed (F_1,23_ = 64.23; *p* < 0.001). Post hoc tests indicated that while the control group displayed no significant changes in 1RM load after the ten-week intervention (*p* = 0.166), the ST group obtained a significant increase (*p* < 0.001; CI-95%: 6.9–11.8 Kg; effect size = 0.74). Similar pattern was observed in Seated Leg Press exercise, in which a significant interaction was found (F_1,23_ = 94.32; *p* < 0.001). Post hoc tests indicated that only the ST group showed a significant increase in 1RM load after the ten-week intervention (*p* < 0.001; CI-95%: 20.6–31.7 Kg; effect size = 0.80).

A significant interaction was also observed in the diastolic blood pressure (F_1,23_ = 7.05; *p* = 0.016). Post hoc tests indicated that while the control group displayed no significant changes in diastolic blood pressure (DBP) after the ten-week intervention (*p* = 0.131), the ST group obtained a significant decrease (*p* = 0.015; CI-95%: -5.8 –-0.8 mmHg; effect size = 0.28). Regarding systolic blood pressure (SBP), an interaction at the significance threshold was observed. However, despite the reduction observed in the ST group, Post hoc tests indicated that there were no significant changes in neither of the groups after the ten-week intervention (ST group–*p* = 0.124; Control group–*p* = 0.224). No interaction was observed in the remaining variables.

With regard to drugs, the predominance of the central and peripheral actions of the drugs used were equally distributed between the groups, both among those elderly women who consumed only one drug (*p* = 1.000), as well as those who consumed a combination of two drugs (*p* = 0.205).

**Fig 1:**
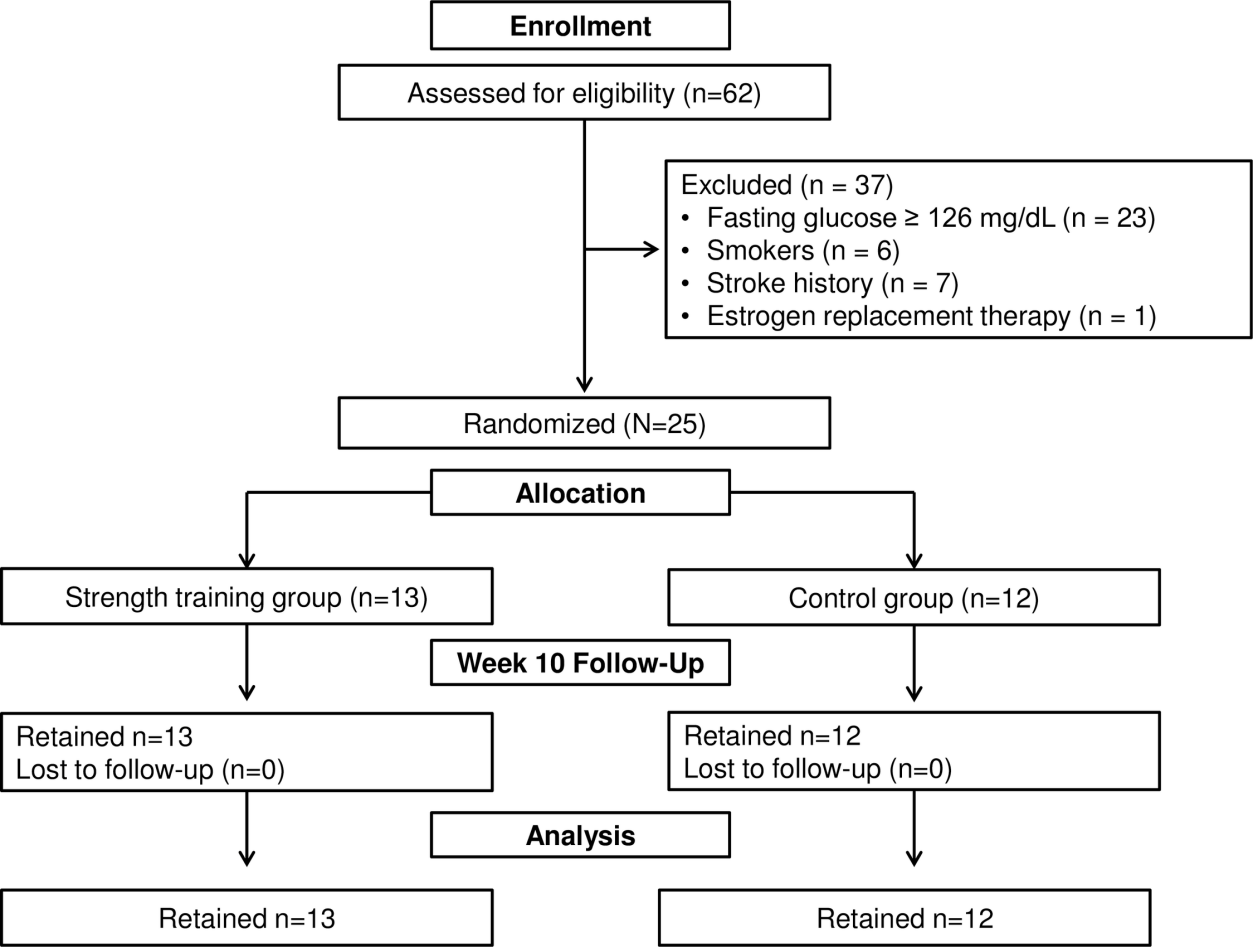
Participant flow through study.

All the HEW allocated to the ST group successfully adapted to the exercise intervention program, or in other words no serious adverse events, such as arrhythmia, muscle injury, hip fracture, or hospitalization related to the exercises were observed, suggesting that ST was safe and within the capabilities of the HEW. Furthermore, no substantial changes were observed in medication or the need for invasive treatment during the study period among those who were analyzed (Fig 1).

### Training effects on blood markers

**Fig 2:**
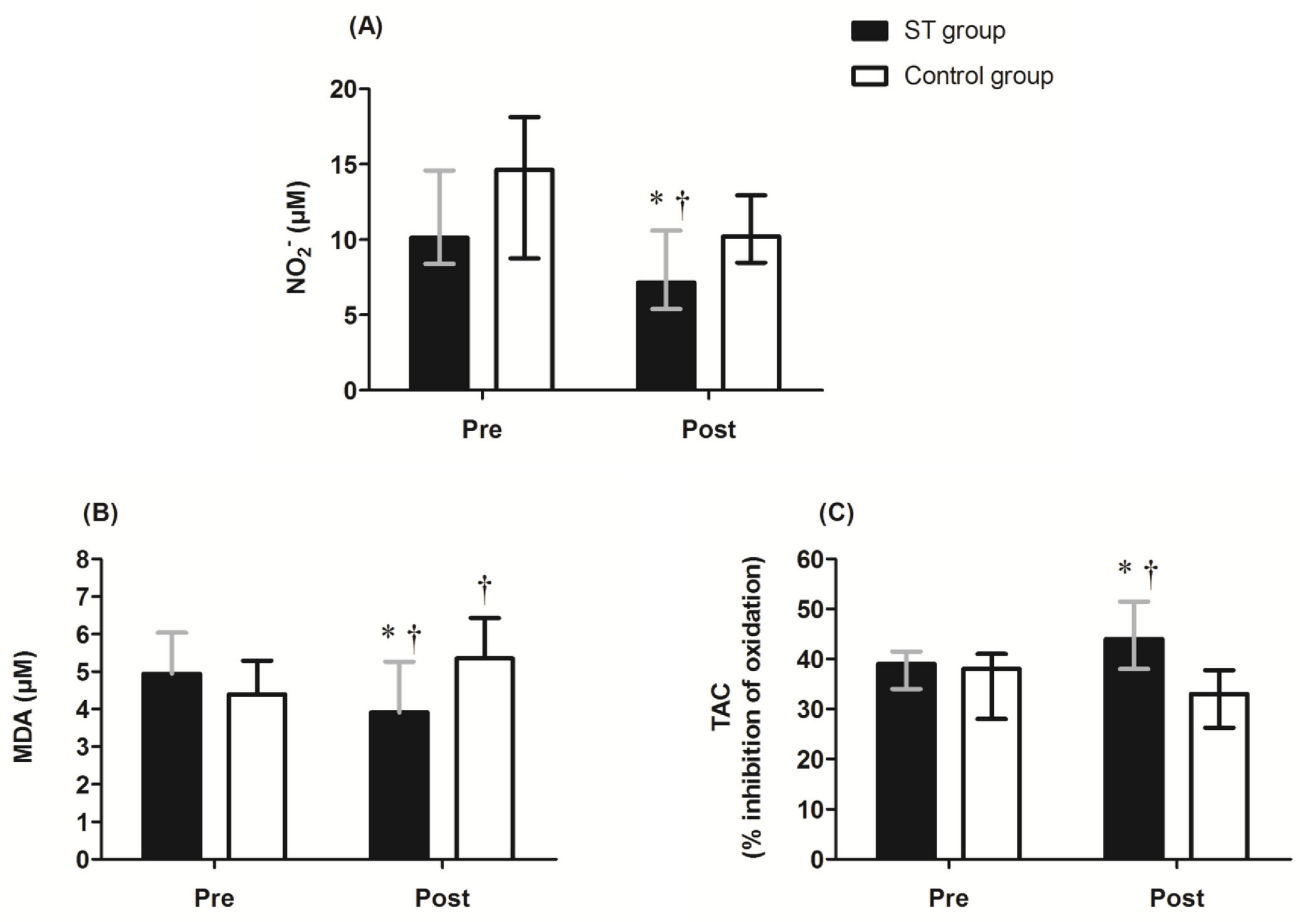
Changes in blood markers of the hypertensive elderly women. *Note*: NO_2_^-^ = plasma nitrite; MDA = plasma malondialdehyde; TAC = total antioxidant capacity. The values in panel A (boxplot A) and C (boxplot C) represent the median and interquartile range. The values in panel B represent the mean and SD. * *p* < 0,05 between groups; † *p* < 0,05 within the group.

Fig 2 shows the changes obtained by the groups for blood markers.

The groups were similar for all blood markers before intervention (p > 0.05). After ten weeks, only the ST group showed a significant reduction in NO_2_^-^ (pre: Md = 10.13; Q_25_ –Q_75_ = 8.40–14.58 μM vs post: Md = 7.16; Q_25_ –Q_75_ = 5.39–10.61; *p* = 0.034) and a significant increase in TAC (pre: Md = 39.0; Q_25_ –Q_75_ = 34.0–41.5% vs post: Md = 44.0; Q_25_ –Q_75_ = 38.0–51.5%; *p* = 0.006).

In relation to MDA, a significant interaction (group*time) was observed (F_1,23_ = 19.46; *p* < 0.001). Post hoc tests revealed that while plasma MDA values significantly increased in the control group after the ten-week intervention (*p* < 0.001), there was a significant reduction in the ST group (**pre:** 4.94 ± 1.10 μM vs **post:** 3.90 ± 1.35 μM; *p* = 0.025; CI-95%: -1.92 –-0.16 μM; effect size = 0.50).

**Fig 3:**
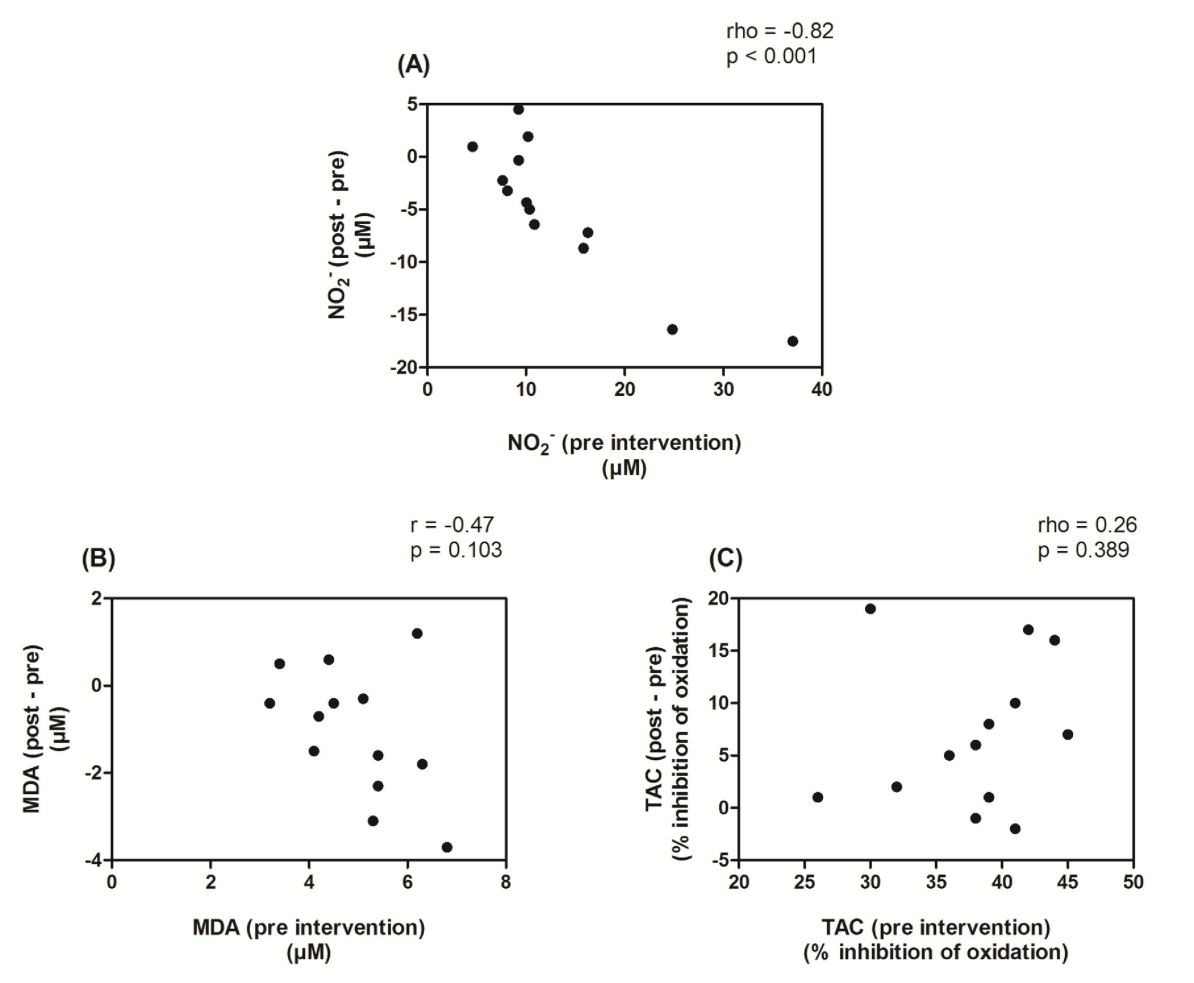
Correlations between changes in the blood markers of the strength training group with their values at the pre-intervention period. *Note*: NO_2_^-^ = plasma nitrite; MDA = plasma malondialdehyde; TAC = total antioxidant capacity. Panel A and C–Spearman’s correlation coefficient; Panel B—Pearson's correlation coefficient.

Fig 3 shows the correlation between changes in the blood markers (post—pre) of the HEW of the ST group and their values at the pre-intervention period.

The only strong and significant correlation can be seen in Panel A, in which the NO_2_^-^ values are present. Although Panel B does not show a significant correlation, it can be seen in both Panel B and A that the greatest reductions in NO_2_^-^ and MDA values were obtained by the HEW with the highest values for the respective markers in the pre-intervention period. The correlation between the TAC values was weak and not significant.

**Fig 4:**
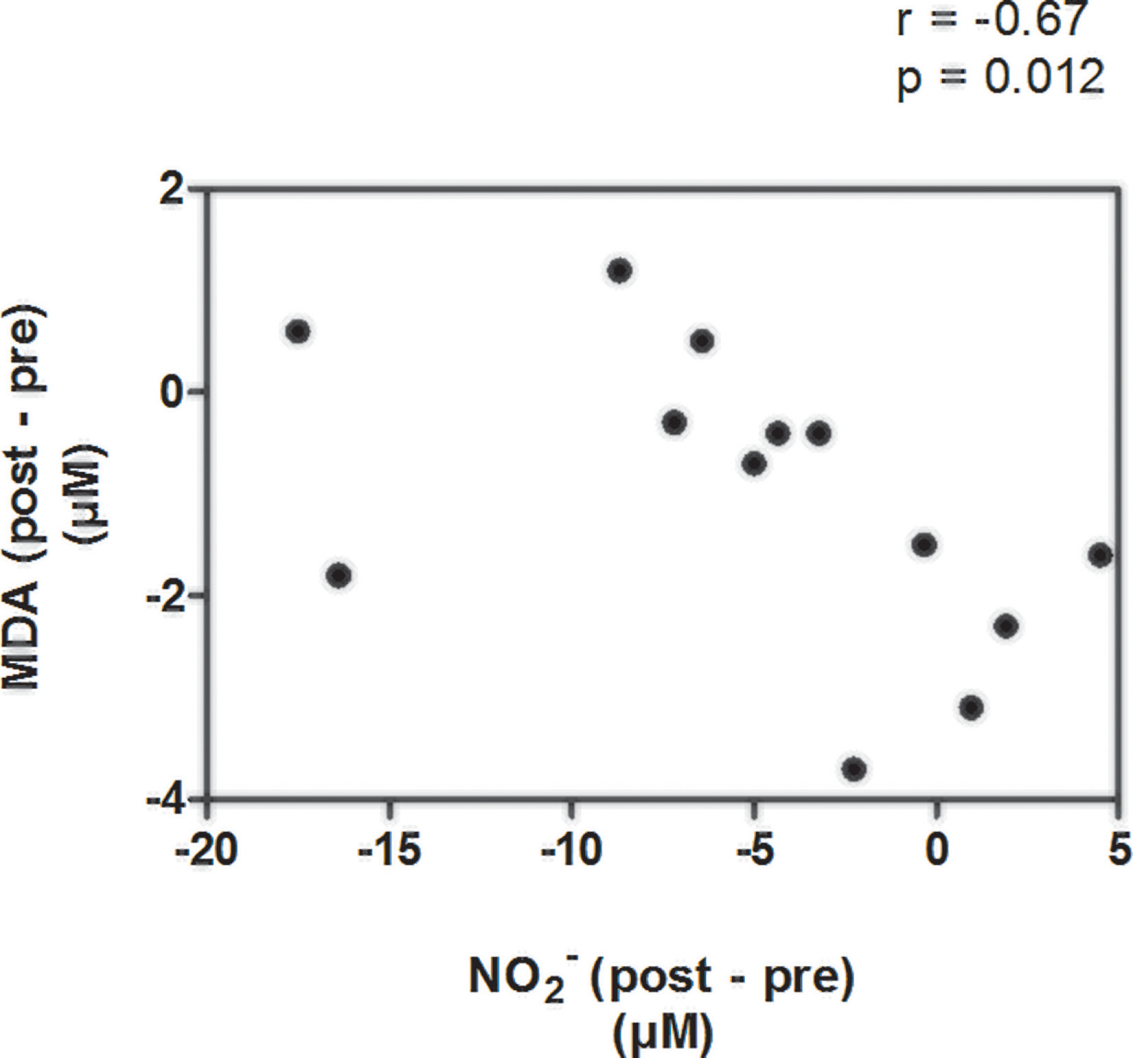
Correlation between changes in the plasma malondialdehyde concentration with changes in the plasma nitrite concentration of the strength training group. *Note*: NO_2_^-^ = plasma nitrite; MDA = plasma malondialdehyde; r = Pearson's correlation coefficient.

The only changes between blood markers which are correlated with each other were among ΔMDA and ΔNO_2_^-^ (Fig 4).

The correlation between ΔMDA and ΔNO_2_^-^ was moderate and significant. Furthermore, it was negative correlation.

### Training effects on forearm vasodilatation and mean blood pressure

Table 2 shows the changes obtained in the groups in forearm vasodilatation and MBP during the experimental protocol.

**Table 2:** Changes in mean blood pressure, forearm blood flow and vascular conductance at basal and during three minutes of static handgrip exercise in the elderly hypertensive women.

		Basal	Minute 1	Minute 2	Minute 3
Mean blood pressure (mmHg)					
ST group	Pre	93.1 ±6.3	95.3 ±7.0	100.7 ±6.4 †	104.5 ±5.8 †
	Post	88.9 ±5.4 *‡	92.9 ±4.7 †‡	95.9 ±4.9 *†‡	99.1 ±4.6 *†‡
Control group	Pre	91.6 ±8.7	94.0 ±6.6	99.5 ±6.0 †	104.9 ±5.6 †
	Post	95.9 ±7.8	99.3 ±8.5 *†	102.8 ±8.3 †	109.4 ±9.7 †
Forearm blood flow (ml.min^-1^.100ml^-1^)					
ST group	Pre	3.29 ±0.71	3.51 ±0.95	3.82 ±1.03 †	4.02 ±1.25 †
	Post	4.62 ±1.07 *‡	4.82 ±1.05 *†‡	5.06 ±1.08 *†‡	5.28 ±1.04 *†‡
Control group	Pre	3.46 ±0.51	3.61 ±0.85	3.80 ±0.85	4.10 ±0.92 †
	Post	3.21 ±0.83	3.23 ±0.89	3.35 ±1.09	3.59 ±1.12
Vascular conductance (units)					
ST group	Pre	3.56 ±0.88	3.71 ±1.07	3.80 ±1.07	3.84 ±1.22
	Post	5.21 ±1.28 *‡	5.21 ±1.21 *‡	5.29 ±1.20 *‡	5.33 ±1.08 *‡
Control group	Pre	3.81 ±0.68	3.85 ±0.85	3.81 ±0.78	3.91 ±0.82
	Post	3.37 ±0.96	3.28 ±0.99	3.29 ±1.15	3.32 ±1.10

* *p* < 0.05 between periods within group

† *p* < 0.05 with respect to basal

‡ p < 0.05 between groups within period.

A significant interaction (group*time) was observed for basal MBP (F_1,23_ = 6.47; p = 0.020). Post hoc tests indicated that while the control group displayed no significant changes in basal MBP after the ten-week intervention (*p* = 0.170), the ST group obtained a significant reduction (Δ = -4.2 ±5.3 mmHg; *p* = 0.035; CI-95%: -8.0 –-0.4 mmHg; effect size = 0.46). Similar pattern was observed in basal FBF and VC, in which a significant interaction was found (**FBF**–F_1,23_ = 14.28; p = 0.001; **VC**–F_1,23_ = 18.91; p < 0.001). Post hoc tests indicated that only the ST group showed a significant increase in the basal FBF and VC after the ten-week intervention (**FBF**–Δ = 1.3 ±0.9 ml.min^-1^.100ml^-1^; *p* = 0.002; CI-95%: 0.62–2.03 ml.min^-1^.100ml^-1^; effect size = 0.59; **VC**–Δ = 1.6 ±1.0 units; *p* = 0.001; CI-95%: 0.93–2.38 units; effect size = 0.63).

During SHE at post intervention period, only the ST group showed a significant reduction in MBP, which was significant from the second minute. In regard to FBF and VC, only the ST group obtained a significant increase for every minute that elapsed during SHE.

### Correlations between blood marker changes with cardiovascular changes

Table 3 shows the correlation between changes in basal VC and MBP with the changes in the blood markers of the ST group.

**Table 3:** Correlations between changes in the basal vascular conductance and mean blood pressure with changes in the blood markers of the strength training group.

	ΔNO_2_^-^	ΔMDA	ΔTAC
**ΔVC**	-0.56*	0.64*	-0.18
**ΔMBP**	-0.41	0.31	0.27

*Note*: ΔVC–basal vascular conductance (post–pre); ΔMBP–basal mean blood pressure (post–pre); ΔNO_2_^-^ - plasma nitrite (post–pre); ΔMDA–plasma malondialdehyde (post–pre); ΔTAC–total antioxidant capacity (post–pre).

* Significant Pearson's correlation coefficient (p < 0.05).

A moderate correlation was observed between changes in basal VC and MBP with the changes in NO_2_^-^ and MDA, which were significant when basal VC was considered (p = 0.047 and p = 0.019 respectively).

**Fig 5:**
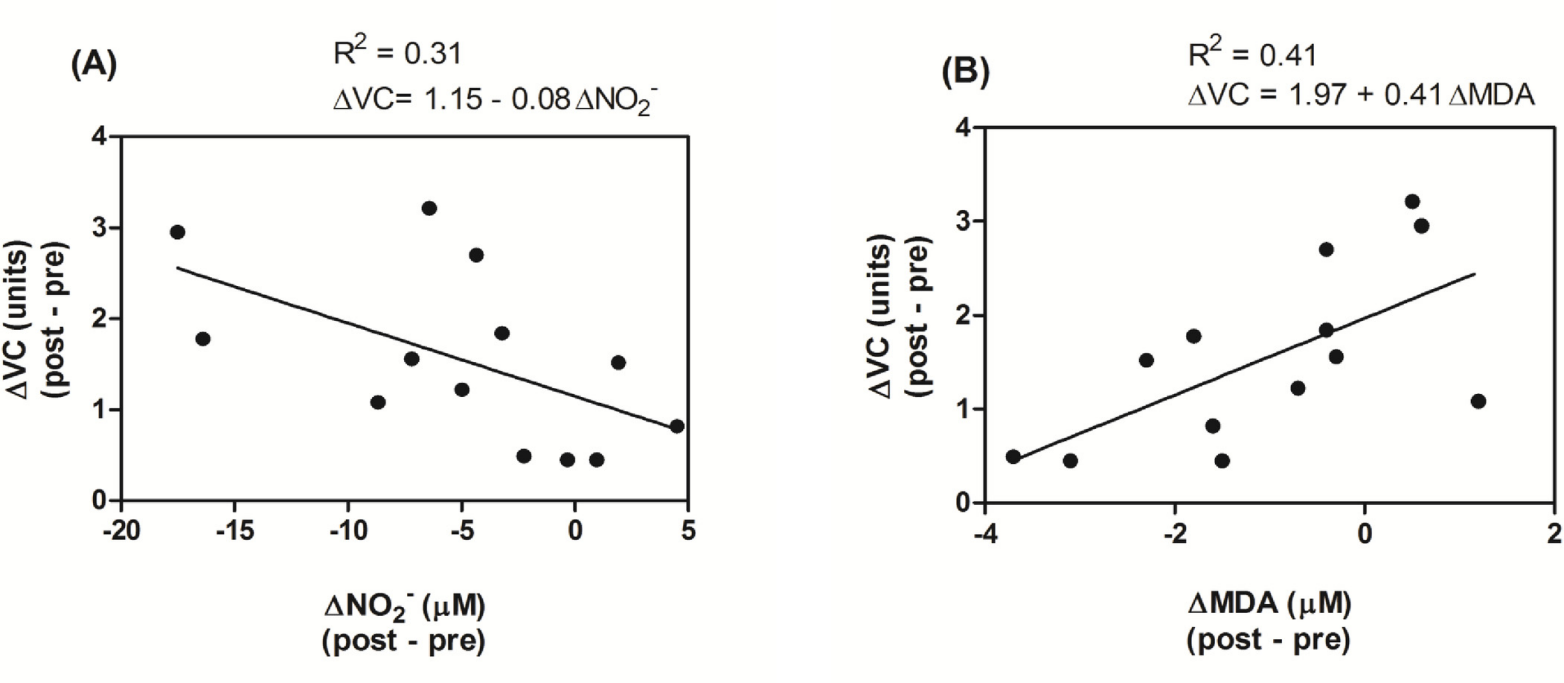
Correlations between changes in the vascular conductance values with the changes in the plasma nitrite and plasma malondialdehyde values. *Note*: ΔCV–basal vascular conductance (post–pre); ΔNO_2_^-^ - plasma nitrite (post–pre); ΔMDA–plasma malondialdehyde (post–pre); Panel A–adjusted R^2^ = 0.25; Panel B–adjusted R^2^ = 0.36.

In Fig 5, using simple linear regression, it can be seen to what extent the changes in NO_2_^-^ and MDA could explain the variability of the changes in VC.

The simple linear regression models presented above demonstrate that ΔNO_2_^-^ and ΔMDA can explain 31% and 41% of ΔCV variation, respectively.

## Discussion

The results of this randomized clinical trial demonstrated that ST was beneficial in reducing oxidative stress, as the ST group achieved a reduction in MDA and an increase in TAC. Cardiovascular benefits were also obtained by the HEW of the ST group, who achieved decrease reduction in MBP and an increase in FBF and VC both at basal condition, as well as during the SHE. When analyzing the relationship between oxidative stress and the cardiovascular variables of the HEW who participated in ST, it was found that NO_2_^-^ and MDA had a moderate correlation with CV and MBP. Such blood markers reduced more significantly in the HEW who had the highest values in the pre-intervention period, and their variations explained a large part of the variation in the ΔVC.

Interestingly, the present study demonstrated that the HEW of the ST group displayed a significant reduction in NO_2_^-^. Although this may appear to contradict findings in literature by representing a decrease in nitric oxide (NO) bioavailability, in fact this represents one of the most remarkable aspects of the NO molecule, which has complex and antagonistic roles, depending on tissue concentrations [31].

Within this context, the NO molecule can act beneficially in the microvascular system, promoting vasodilatation, but, paradoxically, may be involved in the production of cytotoxic radicals. The literature has shown that NO, at high levels, can be converted to pro-oxidant species, such as peroxynitrite (ONNO-), which is considered a potent effector of oxidative stress. Although not addressed here, it has been proposed elsewhere that peroxynitrite might begin the process of lipid peroxidation and potentialize inflammatory injury in vascular cells [32]. The data of the present study indicated that in the pre-intervention period, lipid peroxidation was strongly present in the HEW, due to the higher MDA values observed. Del Rio, Stewart [33] reported that plasma MDA is a major by-product of lipid peroxidation reaction, and is considered an important biological marker of oxidative stress. Therefore, these findings suggest a link between oxidative damage triggered by NO, with the consequently increase in the plasma MDA concentration. However, despite we found the moderate and significant correlation between ΔMDA and ΔNO_2_^-^, it was negative correlation (Fig 4). Probably, this can be explained by the presence of another bioactive lipid peroxidation product, such as 4-Hydroxy-nonenal, which could, possibly, be changing the direction of the correlation [34].

Literature has reported that hypertension among the elderly is associated with high oxidative stress which can be determined by high values of plasmatic MDA [19, 20], and low TAC [35]. Several lines of evidence support the idea that oxidative stress, which develops over advancing age even in healthy elderly persons, may contribute to vascular dysfunction [36–38].

The results of the present study demonstrated that the ST program was able to decrease NO_2_^-^ and MDA plasma concentration, besides increasing TAC, thus reducing the oxidative stress of the HEW. In turn, there was a correlation between this benefit with the results obtained in the CV and basal MBP.

In this sense, it can be suggested that the reduction of oxidant activity (possibly triggered by NO), added to the increased TAC, among the HEW of the ST group, was responsible for obtaining a favorable relationship between oxidant/antioxidant activity. Such alterations can indicate one of the mechanisms involved in the increased forearm vasodilatation and reduced MBP after ten weeks of strength training. In fact, the lower basal vasodilatation and elevated BP values in the hypertensive elderly are related to the endothelial dysfunction promoted by high oxidative stress that is normally present in these individuals [21].

Some previous studies have shown that strength training has a protective effect against lipid peroxidation among the elderly [22, 39] which is mediated by an increase in antioxidant activity [40], especially if the training is performed at lighter intensities.

Vincent, Vincent [22] found that older people who trained at a lower intensity with more repetitions (50% 1RM—13 reps per exercise) experienced a more pronounced protective effect against lipid peroxidation than older people who trained at a higher intensity with fewer repetitions (80% 1RM—8 reps per exercise). Although the authors did not investigate the mechanisms involved in these results, it can be suggested that there was an upregulation in the antioxidant enzymes of elderly persons who trained with a greater number of repetitions. Such speculation is based on the elegant review by Powers and Jackson [41] in which the authors reported that the antioxidant enzyme activity of the superoxide dismutase and catalase results in a more pronounced chronic increase in training programs, in which there is greater activation of the oxidative fibers.

As the ST program applied in this study prioritized lighter intensities, it can be suggested that there was a potentiation of the antioxidant enzymatic effect.

In relation to SHE, cardiovascular benefits were also observed in the HEW in the ST group (FBF increased and MBP decreased). SHE is considered as a sympathoexcitatory maneuver, in which the cardiovascular responses are mediated by activation of the central command and the exercise pressor reflex (EPR) (muscle mechanoreceptors and metaboreceptors) [30]. When the descending and ascending stimuli emitted, respectively, by the central command and EPR, reach the medulla oblongata, a sympathetic efferent discharge is fired, which is responsible for promoting an increase in blood pressure and arterial resistance [42].

Some experimental studies have indicated that EPR sensibility may increase in the presence of hypertension. For example, Leal, Williams [43] demonstrated that spontaneously hypertensive rats had a higher blood pressure response than control rats during procedures which stimulated muscle mechanoreceptors and metaboreceptors. Similar results were also observed in a study by Smith, Williams [44]. The results obtained in experimental animal models have been confirmed in studies with elderly hypertensive patients. In this sense, Delaney, Greaney [45] reported that the high MBP values observed in elderly hypertensive patients who performed SHE were due to an exaggerated sympathetic response during exercise. This exacerbated response was described by Greaney, Matthews [46] as the result of an exaggerated function of the EPR. Sidhu, Weavil [47] found that the pharmacological blockade of the EPR (inhibition of the group III / IV muscle afferent feedback) resulted in increased blood flow in elderly individuals during lower limb exercises. The authors indicated that during exercise with an intact afferent feedback mechanism, group III / IV muscle afferents may hinder blood flow in the elderly.

In addition to EPR, Liang, Mitchell [48] provided the first evidence in literature indicating that changes in the function of the central command (exacerbated activation) can significantly contribute to the generation of an exacerbated cardiovascular response during exercise in hypertensive individuals.

Thus, the reduction in MBP and increase in FBF in the HEW in the ST group during SHE, may have occurred as the result of a decrease in their sympathetic activity, which may have been mediated by reduced activation of the EPR and central command.

The reduction of oxidative stress in the post-intervention period may have contributed to the reduction in the exaggerated function of the EPR and the central command. Hawkins, Leal [49] demonstrated that the reduction of the endogenous activity of the superoxide anion (promoted by increased antioxidant activity), within the nucleus of the solitary tract reduced mechanoreflex hyperactivity and in turn EPR hyperactivity. Additionally, Koba, Hisatome [50] demonstrated that oxidative stress plays a role in sensitizing the rostral ventrolateral medulla neurons which respond to central command activation, thereby exaggerating central command-elicited sympathoexcitation.

EPR, central command and arterial baroreflex are the neural mechanisms most considered in literature, which act by promoting hemodynamic and cardiovascular adjustments during exercise.

Baroreflex dysfunction is an important and common characteristic of hypertension and aging and is closely related to sympathetic hyperactivity [51, 52]. Increased baroreflex sensitivity may have occurred after the ten weeks of ST, which would help to explain the cardiovascular benefits observed during the SHE in the post-intervention period. The decrease in oxidative stress may also have influenced possible increased baroreflex sensitivity because of the existence of a positive feedback between high oxidative stress and baroreflex dysfunction in hypertensive sedentary rats, as Masson, Costa [53] reported in an experimental study.

Based on this information from literature, it can be suggested that the benefits observed in forearm vasodilatation and MBP during SHE are related to the reduction of oxidative stress, which in turn may have contributed to an increase in arterial baroreflex sensitivity, as well as to reducing EPR and central command hyperactivity. All the likely changes taken together, therefore, may have caused a decrease in sympathetic nerve activity and thus promoted the results observed during SHE.

### Limitations

A major limitation of this study was related to the inclusion of hypertensive elderly women who were taking antihypertensive medication with peripheral activity. These drugs promote peripheral vasodilatation. Thus, the benefits of forearm vasodilatation could be attributed to the greater use of this type of medication in the ST group than in the Control group. However, the present study showed that both groups had the same proportion of use of these drugs. Furthermore, the results of the present study were obtained among elderly women who presented normalized blood pressure, due to the use of one or two antihypertensive drugs. It is not known whether the same results would be achieved if the elderly women presented moderate or severe hypertension, situations which require a combination of three or more drugs to normalize blood pressure levels.

### Conclusions and public health implications

The strength training program promoted a reduction in the oxidative stress of the HEW, which in turn, was correlated with their cardiovascular benefits. Such benefits were characterized by an increase in forearm vasodilatation and a reduction in MBP, both at rest and during SHE. However, the results of the correlations do not completely elucidate the mechanisms involved between the cardiovascular improvements with the reduction of oxidative stress in hypertensive elderly women. Based only on these correlations, it is not possible to determine a cause-and-effect relationship.

These findings have a number of implications. Firstly, strength training has been found to be an effective strategy for reducing the blood pressure of the elderly women, in whom the presence of high blood pressure is considered more serious, and less easy to control [10]. Secondly, increased basal FBF and VC is important for better muscle perfusion, a greater supply of oxygen and, consequently, an increase in functional capacity [9]. Thirdly, the reduction of oxidative activity can contribute to reducing mortality in the elderly [24]. Fourthly, the reduction in blood pressure during exercise is clinically important, as increased blood pressure response to exercise is associated with cardiovascular and cerebrovascular events, which can occur during or after exercise [7].

## Supporting Information

S1 File Protocol for Determining Nitrite Concentration. (DOC)

S2 File CONSORT Checklist. (DOC)

S3 File Detailed study protocol (English language). (DOCX)

S4 File Detailed study protocol (original language). (DOCX)

S1 Table Linear reverse periodization of strength training that was held with elderly hypertensive women. PES—Perceived Exertion Scale (*OMNI-RES)* adapted for strength training.
(DOC)
