# Supplementary material for: Effect of Strength Training on Oxidative Stress and the Correlation of the Same with Forearm Vasodilatation and Blood Pressure of Hypertensive Elderly Women: A Randomized Clinical Trial
Source: PLoS One. 2016 Aug 16;11(8):e0161178. doi: 10.1371/journal.pone.0161178 (PMC4986983; doi:10.1371/journal.pone.0161178)
Supplement: S1 File — (DOC) [file pone.0161178.s001.doc]

**Protocol for Determining Nitrite Concentration (PROMEGA®, Griess Reagent System)**

**Materials to Be Supplied by the User**

- reagent reservoirs and multichannel pipettor
- 96-well flat-bottom enzymatic assay plate
- plate reader with 520–550nm filter

1. **Preparation of a Nitrite Standard Reference Curve**

A Nitrite Standard reference curve must be prepared **for each assay** for accurate quantitation of NO2– levels in experimental samples. Prepare reference curve(s) in the same matrix or buffer used for experimental samples.

1. Prepare 1ml of a 100μM nitrite solution by diluting the provided 0.1M Nitrite Standard 1:1,000 in the matrix or buffer used for the experimental samples.
2. Designate 3 columns (24 wells) in the 96-well plate for the Nitrite Standard reference curve (Figure 1). Dispense 50μl of the appropriate matrix or buffer into the wells in rows B–H.
3. Add 100μl of the 100μM nitrite solution to the remaining 3 wells in row A.
4. Immediately perform 6 serial twofold dilutions (50μl/well) in triplicate down the plate to generate the Nitrite Standard reference curve (100, 50, 25, 12.5, 6.25, 3.13 and 1.56μM), discarding 50μl from the 1.56μM set of wells. Do not add any nitrite solution to the last set of wells (0μM).

**Note:** The final volume in each well is 50μl, and the nitrite concentration range is 0–100μM.

| 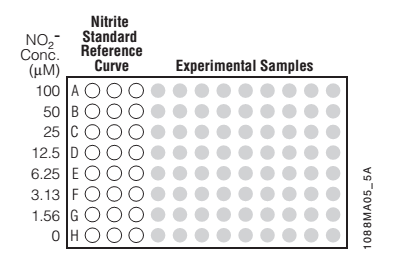 |
| --- |
| **Figure 1.** Suggested plate format for the Nitrite Standard reference curve. |
| **Note:** Source – PROMEGA®, Griess Reagent System. |

1. **Nitrite Measurement (Griess Reaction)**
2. Allow the Sulfanilamide Solution and NED Solution to equilibrate to room temperature (15–30 minutes).
3. Add 50μl of each experimental sample to wells in duplicate or triplicate.
4. Using a multichannel pipettor, dispense 50μl of the Sulfanilamide Solution to all experimental samples and wells containing the dilution series for the Nitrite Standard reference curve.
5. Incubate 5–10 minutes at room temperature, protected from light.
6. Using a multichannel pipettor, dispense 50μl of the NED Solution to all wells.
7. Incubate at room temperature for 5–10 minutes, protected from light. A purple / magenta color will begin to form immediately.
8. Measure absorbance within 30 minutes in a plate reader with a filter between 520nm and 550nm. See Figure 2 for an absorbance spectrum of the colored azo compound.

**Caution:** Measure absorbance within 30 minutes. Color may fade after this time.

| 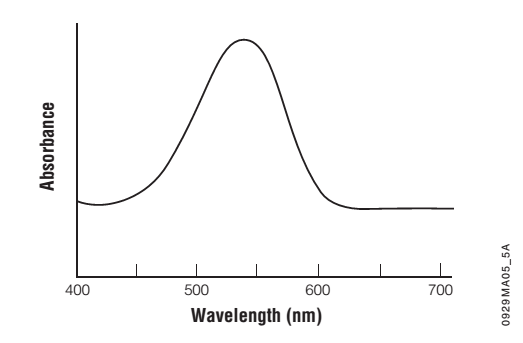 |
| --- |
| **Figure 2.** Absorbance spectrum of the colored azo compound. |
| **Note:** Source – PROMEGA®, Griess Reagent System. |

1. **Determination of Nitrite Concentrations in Experimental Samples**
2. To generate a Nitrite Standard reference curve, plot the average absorbance value of each concentration of the Nitrite Standard as a function of "Y" with nitrite concentration as a function of "X".
3. Determine average absorbance value of each experimental sample. Determine its concentration by comparison to the Nitrite Standard reference curve.
