# Supplementary material for: Effect of Strength Training on Oxidative Stress and the Correlation of the Same with Forearm Vasodilatation and Blood Pressure of Hypertensive Elderly Women: A Randomized Clinical Trial
Source: PLoS One. 2016 Aug 16;11(8):e0161178. doi: 10.1371/journal.pone.0161178 (PMC4986983; doi:10.1371/journal.pone.0161178)
Supplement: S3 File — (DOCX) [file pone.0161178.s003.docx]

**Detailed study protocol (english language)**

**Methods**

Participants

Sedentary hypertensive elderly women were recruited between July 2014 and January 2015 through the Federal University of Paraiba in Brazil, and through media advertising. Using a moderate effect size of f = .50 ([SULLIVAN; FEINN, 2012](#_ENREF_9)) and a power of 80% (for one tailed alpha = .05), which is often recommended as an appropriate power in behavioral research ([GREEN, 1991](#_ENREF_2)), and assuming a moderately strong correlation among the repeated measures (r  =  .50), the power analysis yielded a required sample size of 21 participants. For this two-arm RCT, the target sample size was successfully met and N = 25 participants (mean age = 66.1 ± 5.2 years) were recruited and were randomized into either a 10-week ST intervention or a control group.

The participants were between 60 and 75 years of age, female, had been sedentary for at least the previous 6 months (<2 days per week of structured physical activity, no regular ongoing resistance exercise performance), and were taking medication for hypertension following prior diagnosis, checked by a cardiologist, who provided consent to participate in the 10-week RCT. Participants who presented a history or evidence of hematologic disease, peripheral vascular disease, or strokes, were excluded from the study. Other exclusion criteria were considered, such as: fasting plasma glucose ≥ 126 mg/dL, if the individual smoked or consumed more than 60 g of ethanol (corresponding to half a liter of wine) per day, and elderly women who were undergoing estrogen replacement therapy.

Procedures

The general characteristics data and all baseline assessments were conducted prior to randomizing the participants into the ST intervention or control group. Both assessors and participants were blinded regarding group placement at the time of the baseline assessments. Figure 1 shows the flow of participants through the study following baseline testing and randomization.

| 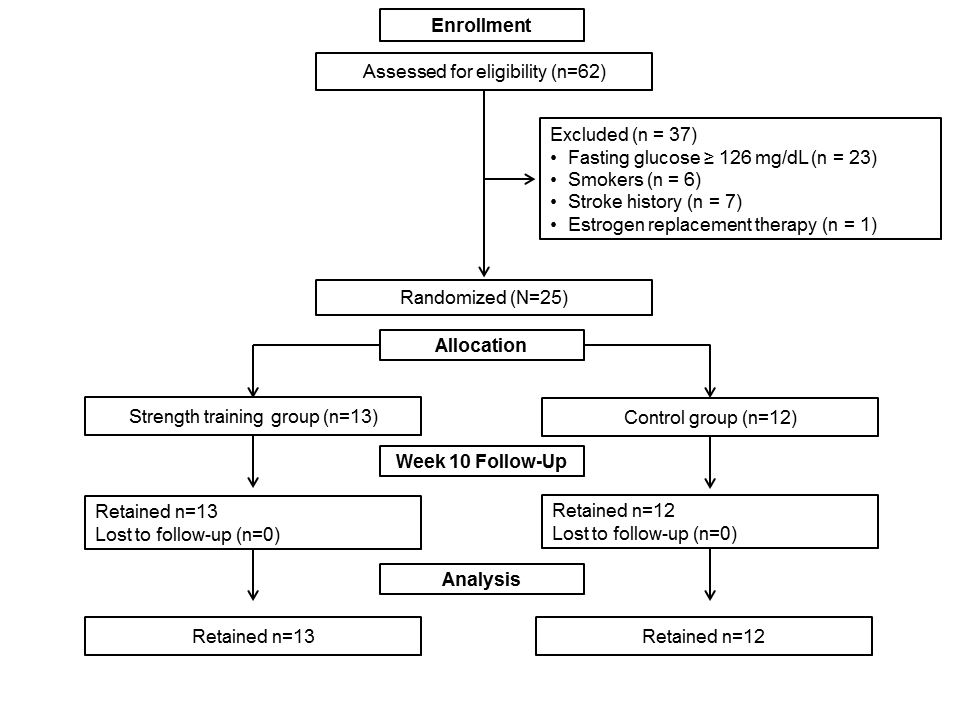 |
| --- |
| **Figure 1.** Flow of participants through the study. |

General characteristics data was collected in the first meeting with the participants. Subsequently, the volunteers were informed about the need to carry out fasting for at least 12 hours, so that blood could be collected on the following day for laboratory tests. Once the results of the blood tests had been obtained, the exclusion criteria were applied. A date was scheduled for the elderly women who were not excluded assess basal vasodilatation and the vasodilatory response.

Such response was checked through alterations in forearm blood flow in different experimental conditions (described below). Such changes were evaluated by venous occlusion plethysmography. Before collection of these results, the elderly women were advised not to perform any kind of exercise on the day before the procedure. It was also recommended that they keep to their usual alimentary habits, and avoid ingesting stimulant drinks, such as coffee, chocolate, soda or alcohol.

**Strength Training Group**

The ST intervention was led by physical education professionals with experience in the ST field. The supervised group sessions were held twice a week for five weeks, with frequency increasing to three times thereafter. The exercise techniques were explained to participants in two training sessions 48 hours apart, carried out prior to the beginning of the training program. These sessions were intended to make the participants familiar with the exercises. Ten repetitions of each exercise were performed during the familiarization sessions, using the lightest possible load of each device, so teaching the elderly women to adopt a proper body positioning, range of motion (considering their individual limitations), as well as a proper breathing pattern while performing exercises (avoiding the Valsalva maneuver, namely inhaling in the relaxation phase and exhaling in the contraction phase). Additionally, these sessions also had the purpose of teaching the proper interpretation of the scale of perceived exertion (OMNI-RES), adapted for ST ([ROBERTSON et al., 2003](#_ENREF_8)), so that the intensity of the training of the participants could be properly monitored. To facilitate understanding of this subjective scale, the 1RM (maximal repetition) test was applied to the Bench Press and Seated Leg Press exercise, according to previously described protocols ([KRAEMER et al., 1995](#_ENREF_4)). This procedure also served to verify the training efficiency in relation to strength gains.

The training program consisted of nine exercises, which were carried out in the following order: Seated Leg Press; Seated Row Machine; Trunk Flexion; Knee Flexion Machine, Bench Press, Trunk Extension Machine, Push Press, Standing Plantar Flexion and Front Pulldown. All the variables related to the strength training protocols applied in this study can be seen in the Table 1.

| **Table 1.** Linear reverse periodization of strength training that was held with elderly hypertensive women. | | | | | |
| --- | --- | --- | --- | --- | --- |
| Week | Weekly Frequency | Sets | Repetition | Rest between sets | PES* |
| 1 | 2 | 1 | 9 – 11 | 120 seconds | 5 – 7 |
| 2 | 2 | 1 | 9 – 11 | 120 seconds | 5 – 7 |
| 3 | 2 | 2 | 9 – 11 | 120 seconds | 5 – 7 |
| 4 | 2 | 2 | 9 – 11 | 120 seconds | 5 – 7 |
| 5 | 2 | 2 | 11 – 13 | 90 seconds | 5 – 7 |
| 6 | 3 | 2 | 11 – 13 | 90 seconds | 5 – 7 |
| 7 | 3 | 2 | 11 – 13 | 90 seconds | 5 – 7 |
| 8 | 3 | 3 | 11 – 13 | 90 seconds | 5 – 7 |
| 9 | 3 | 3 | 13 – 15 | 60 seconds | 5 – 7 |
| 10 | 3 | 3 | 13 – 15 | 60 seconds | 5 – 7 |
| PES* - Perceived Exertion Scale (*OMNI-RES)* adapted for strength training. | | | | | |

**Control Group**

The hypertensive elderly women allocated to the control group performed that the same assessments as the ST group. The only difference was that they remained sedentary during the 10-week follow-up, when they were advised to maintain their usual habits. For ethical reasons, after the follow-up period, they were invited to participate in the training program.

Measures

In addition to general characteristics data, the following assessments were completed by all participants pre and post intervention.

**Vasodilatation**

Evaluation of basal vasodilatation and the vasodilatory response (vasodilatation during static handgrip exercise and reactive hyperemia) was performed after the participant confirmed that she had complied with the recommendations. When confirmation was given, the elderly person was initially submitted to the maximal voluntary contraction test (MVC) using the handgrip. This test was used to determine the load of the static handgrip exercise (SHE) (described later). After application of this test, the elderly person was placed in the supine position on a stretcher, in order for the equipment used for assessment of forearm blood flow to be fitted. This was measured by venous occlusion plethysmography. The methodological procedures of venous occlusion plethysmography have been described elsewhere ([LEKAKIS et al., 2011](#_ENREF_5)). Additionally after placement of the instruments related to the plethysmograph, a pressure cuff (DIXTAL Medical^®^) was placed on the lower third of the elderly leg. The function of this cuff was to record blood pressure (systolic, diastolic and mean), every minute during the analysis of forearm blood flow with the aim of collecting information about vascular conductance (VC) ([blood flow / mean blood pressure] x 100).

Following the procedures related to the MVC test and instrumentation, the elderly person remained lying on the stretcher for 10 minutes until she had returned to normal resting condition. After this period, the records of forearm blood flow were initiated. Such records were made as follows:

**1. Basal:** During the initial instrumentation, as well as placement of the materials related to plethysmography, in the non-dominant arm, surface electrodes were placed on the chest (bipolar positions – derivation D2) to capture the electrocardiographic signal. Therefore, electrocardiogram (ECG) and forearm blood flow were recorded for three minutes.

**2. Static handgrip exercise:** After basal measurements, SHE of the dominant arm was performed for three minutes, at an intensity corresponding to 30% MVC. This maneuver has the ability to promote muscle sympathetic nerve activity (MSNA), which is mediated by central command and the exercise pressor reflex (muscle mechanoreceptors and metaboreceptors) ([MARK et al., 1985](#_ENREF_6)).

# 3. Reactive hyperemia: Ten seconds before the end of the SHE, circulation to the exercising forearm was arrested by inflating the upper arm occlusion cuff (210 mmHg) for three minutes. The purpose of this experiment was to determine the magnitude of change in forearm blood flow, VC and MBP during isolated metaboreflex activation. In this maneuver, blood flow (BF) was assessed in two manners: peak blood flow (pBF) and percent change in blood flow (%BF). The pBF value was obtained during the first three heart beats, in the first wave generated after cuff release. The %BF was calculated for each elderly woman as ((peak blood flow minus the baseline blood flow)/baseline blood flow) * 100 ([JASPERSE et al., 2015](#_ENREF_3)).

The entire schematic model, related to the evaluation of basal vasodilatation and vasodilatory response, can be seen in Figure 2.

| 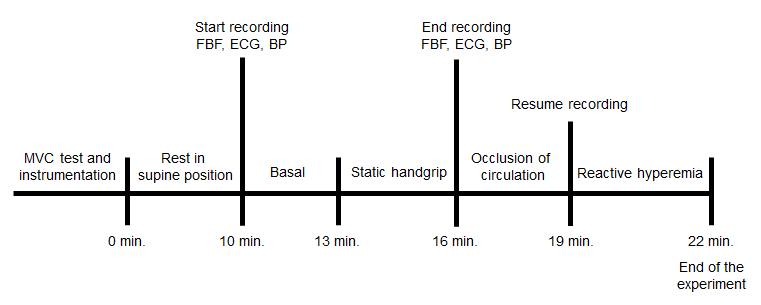 |
| --- |
| **Figure 2.** The timeline of the experimental protocol for assessing basal vasodilatation and vasodilatory response. |
| *Note:* BP – Blood pressure, ECG – electrocardiogram, FBF – forearm blood flow, MVC – maximal voluntary contraction. |

**Analysis of biochemical parameters**

The glucose, triglycerides, HDL - cholesterol and hs-CRP values were measured with a LabMax 240^®^ device, following the standard operating procedure established for biochemical evaluation of each measure and the proper use of reagents. The value of LDL - cholesterol was calculated by the Friedewald formula (LDL-C = total cholesterol - HDL-C - [Triglycerides / 5]).

**Evaluation of nitric oxide bioavailability**

Nitric oxide (NO) bioavailability was evaluated by measurement of plasma nitrite (NO_2_^-^) which was performed by a spectrophotometric procedure based on the Griess reaction. The Griess Reagent System is based on the chemical reaction, which uses sulfanilamide and N-1-napthylethylenediamine dihydrochloride (NED) under acidic (phosphoric acid) conditions.

**Evaluation of oxidative stress**

Evaluation of oxidative stress was conducted by analyzing the plasma malondialdehyde (MDA) and total antioxidant capacity (TAC). The oxidant activity of MDA was quantified by thiobarbituric acid reaction with the decomposition products of hydroperoxide. For more details see reference ([OHKAWA; OHISHI; YAGI, 1979](#_ENREF_7)). The results for TAC were described as percentage of antioxidant activity (%TAC). For details about the procedures, see reference ([BRAND-WILLIAMS; CUVELIER; BERSET, 1995](#_ENREF_1)).

Data Analysis

Data was analyzed using SPSS statistical software with an intention-to-treat approach. The verification of data normality was assessed by the Shapiro-Wilk test. The variables with normal distribution were evaluated by Split-Plot ANOVA (SPANOVA) mixed design, a mixed generalized statistical model that considers an independent factor (group - training vs control) and a dependent or correlated factor (time - pre vs post). If significant interactions were found (group*time), post hoc tests were applied to the comparison pairs, through the Student T test for dependent (intragroup) and independent samples (intergroup). When the analysis in question involved multiple comparisons, the *p* values were submitted to Bonferroni correction. For those variables without normal distribution, the evaluation was conducted by the Wilcoxon test (intragroup), and the U Mann-Whitney test (intergroup).

The effect size (ES) was calculated for the main dependent variables (BF and VC), in order to assess the magnitude of the difference between groups (training vs control), in the post-intervention period, using the following formula (mean difference between groups / pooled standard deviation). The classification of the ES was determined as follows: 0.2 (low); 0.5 (moderate); 0.8 (high); 1.3 (very high).

The correlations between changes in basal BF (ΔBF) and VC (ΔVC) with changes in blood variables (Δhs-CRP, ΔNO_2_^-^, ΔMDA and ΔTAC) were analyzed using Pearson's correlation coefficient (r) when both had normal distribution. Otherwise, the Spearman correlation coefficient (rho) was applied. Such correlations were considered in the ST group using the following formula (Δ = post-training value – pre-training value). To assess the strength of the correlation between variables the following values were considered: 0.2 (weak); 0.5 (moderate); 0.8 (strong).

In addition, comparison of proportions for the use of different types of antihypertensive drugs, consumed by hypertensive elderly women, was performed by Fisher's exact test. In all situations, a significance value less than 5% was considered.

**References**

BRAND-WILLIAMS, W.; CUVELIER, M.; BERSET, C. Use of a free radical method to evaluate antioxidant activity. **LWT-Food Science and Technology,** v. 28, n. 1, p. 25-30, 1995.

GREEN, S. B. How many subjects does it take to do a regression analysis. **Multivariate behavioral research,** v. 26, n. 3, p. 499-510, 1991.

JASPERSE, J. L. et al. Positional differences in reactive hyperemia provide insight into initial phase of exercise hyperemia. **J Appl Physiol (1985)**, p. jap.01253.2013, Jul 2 2015.

KRAEMER, W. J. et al. Strength testing: development and evaluation of methodology. **Physiological assessment of human fitness,** v. 2, p. 119-150, 1995.

LEKAKIS, J. et al. Methods for evaluating endothelial function: a position statement from the European Society of Cardiology Working Group on Peripheral Circulation. **Eur J Cardiovasc Prev Rehabil,** v. 18, n. 6, p. 775-89, Dec 2011.

MARK, A. L. et al. Microneurographic studies of the mechanisms of sympathetic nerve responses to static exercise in humans. **Circ Res,** v. 57, n. 3, p. 461-9, Sep 1985.

OHKAWA, H.; OHISHI, N.; YAGI, K. Assay for lipid peroxides in animal tissues by thiobarbituric acid reaction. **Anal Biochem,** v. 95, n. 2, p. 351-8, Jun 1979.

ROBERTSON, R. J. et al. Concurrent validation of the OMNI perceived exertion scale for resistance exercise. **Med Sci Sports Exerc,** v. 35, n. 2, p. 333-41, Feb 2003.

SULLIVAN, G. M.; FEINN, R. Using Effect Size-or Why the P Value Is Not Enough. **J Grad Med Educ,** v. 4, n. 3, p. 279-82, Sep 2012.
