# Supplementary material for: Effect of Strength Training on Oxidative Stress and the Correlation of the Same with Forearm Vasodilatation and Blood Pressure of Hypertensive Elderly Women: A Randomized Clinical Trial
Source: PLoS One. 2016 Aug 16;11(8):e0161178. doi: 10.1371/journal.pone.0161178 (PMC4986983; doi:10.1371/journal.pone.0161178)
Supplement: S4 File — (DOCX) [file pone.0161178.s004.docx]

**Detailed study protocol (original language)**

**Original title in portuguese language**

**Tese de doutorado:** Efeito do treinamento de força na resposta vasodilatadora de idosas hipertensas: mecanismos relacionados.

**Title in english language**

**Doctoral thesis:** Effect of strength training on vasodilatory response in hypertensive elderly women: related mechanisms.

**Complete and detailed plan for the conduct and analysis of the trial that the ethics committee approved before the trial began (portuguese language)**

**PROCEDIMENTOS METODOLÓGICOS**

**Participantes**

Os sujeitos incluídos neste ensaio clínico controlado e randomizado foram recrutados, após divulgação na mídia a respeito do projeto de pesquisa. Para tanto, foram adotados os seguintes critérios de inclusão:

1. Ter idade entre 60 e 75 anos;
2. Gênero feminino;
3. Não ter praticado nenhum tipo de exercício físico regular (≥ 2 dias por semana) num período de 6 meses anteriores ao início do estudo;
4. Hipertensas medicadas que apresentassem diagnóstico prévio de hipertensão, conferido por um cardiologista.

Dentre as idosas elegíveis para a pesquisa, foram adotados os seguintes critérios de exclusão: apresentar histórico ou evidência de doença hematológica, doença vascular periférica, ou acidente vascular encefálico (AVE). Adicionalmente, foram excluídas do estudo as idosas que, após um período de jejum caracterizado pela ausência de ingestão calórica por pelo menos 12 horas, apresentassem glicose plasmática ≥ 126 mg/dL ([ZITKUS, 2014](#_ENREF_18)). Além disso, também foram excluídas as idosas fumantes, consumidoras de mais de 60g de etanol (correspondente a meio litro de vinho) por dia e aquelas idosas que, porventura, estivessem fazendo terapia de reposição de estrogênio.

As idosas que se interessaram em participar desse estudo e atenderam aos critérios estipulados, foram divididas, aleatoriamente, em dois grupos: Grupo Treinamento e Grupo Controle. Ambos os grupos foram avaliados nos períodos pré e pós-intervenção, com relação às variáveis investigadas nessa pesquisa. Sendo assim, seguem abaixo os detalhamentos de cada procedimento experimental.

**Procedimentos de coleta de dados e instrumentos de medida**

Esse estudo foi previamente aprovado pelo Comitê de Ética em Pesquisa do Centro de Ciências da Saúde da Universidade Federal da Paraíba – CEP/CCS (Protocolo 0216/14 – CAAE:30878514.0.0000.5188). No primeiro dia da semana inicial da pesquisa, foi apresentado e assinado o termo de consentimento livre e esclarecido, conforme resolução 466/12 – CONEP e, em seguida, foi aplicado um questionário previamente elaborado (APÊNDICE I), a fim de coletar informações sobre características demográficas, antropométricas, histórico de doenças e dados relacionados aos hábitos e estilo de vida das idosas.

Posteriormente, foram fornecidas informações às idosas, a respeito da necessidade de realizar um jejum por pelo menos 12 horas, afim de que no dia seguinte fosse feita a coleta sanguínea para os exames laboratoriais. A coleta sanguínea foi realizada por técnicos de laboratório preparados para essa função, os quais coletaram uma amostra de sangue venoso, através de uma seringa descartável, sendo realizada assepsia local com álcool a 70%. Após esse procedimento, foram seguidas as recomendações de armazenamento da amostra, sugeridas por [LIMA et al. (1992)](#_ENREF_8).

Após os resultados dos exames, foi agendado outro dia para avaliação do FS no antebraço das idosas que atenderam aos critérios exigidos (tópico 3.1). Para proceder com a coleta dessa variável, foi, previamente, recomendado às idosas que não realizassem nenhum tipo de exercício físico no dia anterior aos procedimentos. Adicionalmente, elas foram instruídas para que, na noite anterior, mantivessem seus hábitos alimentares usuais, contudo sem ingerir bebidas estimulantes como café, chocolate, refrigerante ou álcool. Além disso, também foi solicitado que elas esvaziassem a bexiga na hora da coleta, caso fosse necessário. Sendo assim, ao observar essas recomendações, a idosa chegava ao laboratório e, inicialmente, era submetida ao teste de contração voluntária máxima (CVM) na preensão manual, com a utilização do dinamômetro Jamar® modelo 5030J1. Tal procedimento foi conduzido, conforme o protocolo adotado por [COLDHAM; LEWIS; LEE (2006)](#_ENREF_4). Esse teste foi utilizado para determinar a carga no EIPM (descrito posteriormente). Após a aplicação desse teste, a idosa foi posicionada em decúbito dorsal, sob uma maca, para ser instrumentada com os equipamentos utilizados para avaliação do FS no antebraço o qual foi aferido pela técnica de pletismografia com oclusão venosa.

A plestimografia com oclusão venosa consiste em colocar um tubo silástico preenchido com mercúrio, conectado a um transdutor de baixa pressão, posicionando-o ao redor do antebraço, a 5 centímetros de distância distal à articulação úmero-radial e conectado a um pletismógrafo (Hokanson®/EC6, Pletysmograph). Um manguito foi colocado ao redor do pulso e outro na parte superior do braço. O manguito de pulso foi inflado a um nível suprassistólico, um minuto antes de se iniciarem as medidas. Em intervalos de dez segundos, o manguito do braço foi inflado, acima da pressão venosa, durante o tempo decorrido em cada medida experimental. O aumento em tensão no tubo silástico refletiu o aumento de volume do antebraço e, consequentemente, a vasodilatação. O sinal da onda do FS foi adquirido de forma “on line”, em um computador, por meio do programa WINDAQ DI200, numa frequência de 500Hz ([LUZ; LAURINDO; CHAGAS, 2003](#_ENREF_9)). Vale ressaltar que, após a colocação dos aparelhos relacionados ao pletismógrafo, foi colocado no terço inferior da perna da idosa, um manguito de pressão (DIXTAL MEDICAL^®^). Esse manguito teve a função de registrar os valores de pressão arterial (sistólica, diastólica e média), minuto a minuto, durante a análise do FS, no intuito de coletar informações a respeito da CV ([fluxo sanguíneo / pressão arterial média] x 100).

Logo em seguida aos procedimentos relacionados ao teste de CVM e de instrumentação, a idosa permaneceu por 10 minutos deitada na maca do laboratório para retornar à condição normal de repouso. Após esse período, foram iniciados os registros do FS no antebraço. Tais registros foram feitos nas seguintes condições experimentais:

1 – Medida basal: durante a instrumentação inicial do procedimento experimental, além da colocação da pletismografia com oclusão venosa no antebraço não – dominante, conforme técnica descrita anteriormente, foram colocados os eletrodos de superfície no tórax da idosa, nas posições bipolares, na derivação DII, para captação do sinal eletrocardiográfico. Dessa forma, foram registrados os sinais basais eletrocardiográficos e de FS no antebraço, por um período de três minutos;

2 – Exercício isométrico de preensão manual (EIPM): após o período basal, foi realizado EIPM com a mão dominante, no intuito de determinar a magnitude das mudanças no FS e na CV do antebraço avaliado, assim como, da pressão arterial média (PAM), durante essa manobra de ativação do comando central, mecanorreceptores e metaborreceptores (manobra simpatoexcitatória) ([RONDON et al., 2006](#_ENREF_14)). Para determinar a carga utilizada, durante esse procedimento, foi considerado o teste de CVM feito previamente, para adotar uma intensidade de 30% da carga máxima obtida. Durante três minutos de ação isométrica, estavam sendo registrados os sinais eletrocardiográficos e de FS, no antebraço cuja mão não estava realizando o exercício. As avaliadas foram instruídas a respirarem normalmente para evitar a realização da manobra de Valsava;

3 – Manobra de oclusão na artéria braquial: dez segundos antes da finalização do EIPM, a circulação sanguínea, no braço cuja mão estava realizando o referido exercício, foi ocluída com o manguito do esfigmomanômetro, inflado para pressão de 210 mmHg por 3 minutos. Durante esse período, não foi realizado nenhum registro. Após os três minutos de oclusão, o manguito foi liberado (hiperemia reativa), como também, retomado os registros eletrocardiográficos e do FS. Esses registros foram ativados por três minutos, com o intuito de avaliar as mudanças no FS, na CV, e na PAM, após uma manobra isolada de ativação do metaborreflexo muscular ([NEGRÃO et al., 2001](#_ENREF_11); [TROMBETTA et al., 2003](#_ENREF_16)). Na hiperemia reativa, o FS foi avaliado de duas maneiras: pico do fluxo sanguíneo (pFS) e o percentual de mudança do fluxo sanguíneo (%FS). A análise do pFS foi referente ao valor do fluxo correspondente aos três primeiros batimentos da primeira onda gerada, após a liberação do manguito. O %FS foi calculado para cada idosa, através da seguinte fórmula: ((pico do fluxo sanguíneo – fluxo sanguíneo basal) / fluxo sanguíneo basal) * 100 ([JASPERSE et al., 2015](#_ENREF_6)).

Todo o modelo esquemático do protocolo experimental de avaliação do FS, nas idosas dessa pesquisa, pode ser visualizado na Figura 1.

| **Figura 1** – Linha do tempo do protocolo experimental de avaliação do fluxo sanguíneo. |
| --- |
| 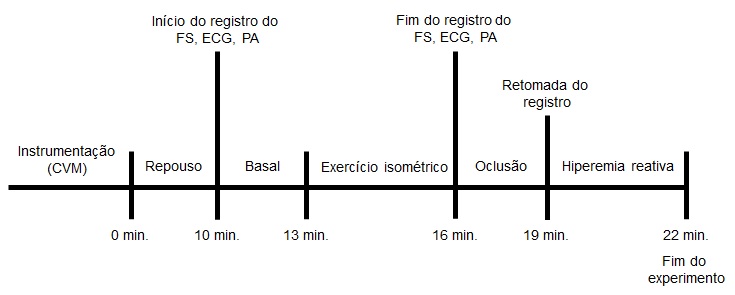 |
| CVM – Contração voluntária máxima; PA – Pressão arterial; FS – Fluxo sanguíneo; ECG – Eletrocardiograma. |

Análise dos parâmetros bioquímicos

Os valores da glicose, triglicérides, HDL – colesterol e da PCRu foram mensurados no aparelho Labmax 240®, tendo o cuidado de cumprir com o procedimento operacional padrão (POP) estabelecido para avaliação de cada medida bioquímica, além da utilização adequada dos respectivos reagentes (LABTEST DIAGNÓSTICA S/A). O valor do LDL – colesterol foi calculado pela fórmula de Friedewald (LDL-C = Colesterol total – HDL-C – [Triglicérides/5]) ([FRIEDEWALD; LEVY; FREDRICKSON, 1972](#_ENREF_5)).

Avaliação da biodisponibilidade de óxido nítrico

A avaliação da biodisponibilidade do NO foi feita, através da dosagem do nitrito plasmático (NO_2_^-^), a qual foi realizada por procedimentos espectrofotométricos baseados na reação de Griess ([BREDT; SNYDER, 1994](#_ENREF_2)). Nesse método, o nitrito reage com a sulfonilamida, em condições ácidas, formando um diazo que se combina com NED (N-(1-naftil)-etilenodiamina- dihidroclorido), que por sua vez forma um cromóforo de cor rósea, com o pico de absorbância em 560nm.

Para proceder com essa reação, foram utilizados três reagentes, através dos seguintes procedimentos: 1. Ácido Fosfórico 5% - 5 ml desse reagente foi colocado no balão volumétrico de 100 ml, completando lentamente o restante do volume com água destilada; 2. Sulfonilamida 1% em ácido fosfórico 5% - foi pesado 0,1g da sulfonilamida que teve 10 ml de ácido fosfórico 5% adicionados ao seu volume; 3. NED 0,1% - foi pesado 10 mg do NED que teve 10 ml de água destilada adicionados ao seu volume. A reação foi controlada pelo tempo, e o produto foi determinado entre 10 min e 2 h, após a mistura dos reagentes. Sendo assim, para gerar uma curva de referência padrão do NO_2_^-^, teve que ser plotado o valor médio da absorbância de cada concentração do NO_2_^-^ padronizado, como uma função do eixo “y”, e a concentração de NO_2_^-^, como uma função do eixo “x”. Portanto, após determinação do valor médio da absorbância de cada amostra experimental, foi determinada a concentração do NO_2_^-^ pela comparação com a curva de referência padrão do NO_2_^-^ (PROMEGA®, Griess Reagent System).

Avaliação do estresse oxidativo

A avaliação do estresse oxidativo foi conduzida, através da análise do MDA plasmático e da capacidade antioxidante total (CAT).

A atividade oxidante do MDA foi quantificada por meio da reação do ácido tiobarbitúrico (TBARS) com os produtos de decomposição dos hidroperóxidos, conforme método descrito por [OHKAWA; OHISHI; YAGI (1979)](#_ENREF_12). Para isso, 250 µl de amostra foram adicionados a cloreto de potássio (KCL) e incubados, em banho maria a 37°C por 60 minutos. Em seguida, a mistura foi precipitada com ácido perclórico (AA) 35% e centrifugado a 14000 rpm por 10 minutos a 4°C. O sobrenadante foi transferido para novos ependorfs e foram adicionados 400 µl de TBARS a 0,6% e incubado a 95 – 100° C por 30 minutos. Após o resfriamento, o material foi lido em espectrofotômetro a um comprimento de onda de 532 nm.

A CAT foi analisada, através do procedimento baseado no método descrito por [BRAND-WILLIAMS; CUVELIER; BERSET (1995)](#_ENREF_1) na qual uma alíquota de 1,25 mg de 2,2 diphenyl-1-picrylhydrasyl (DPPH) foi diluída em 100 mL de etanol (álcool etílico absoluto 99,5%), mantida sob refrigeração e protegida da luz (com papel alumínio ou vidro âmbar). Em tubos apropriados para centrífuga, foram adicionados 3,9 mL da solução de DPPH e, em seguida, acrescentaram-se 100 µL do plasma. Os tubos foram agitados no vórtex e deixados em repouso por 30 minutos. Em seguida, foram centrifugados a 10.000 rpm à temperatura de 20ºC por 15 minutos e o sobrenadante utilizado para a realização da leitura em espectrofotômetro a 515 nm. Os resultados foram expressos em percentual da CAT, onde: CAT = 100 – [DPPH•R]_t_ /[DPPH•R]_B_ 100); sendo [DPPH•R]_t_ e [DPPH•R]_B_ a concentração de DPPH remanescente após 30 minutos, avaliadas na amostra (t) e no branco (B) preparado com água destilada.

**Intervenção**

A intervenção foi conduzida por profissionais formados em Educação Física e só foram avaliadas as idosas que cumpriram com uma frequência mínima de 85% das sessões de treino programadas.

As idosas que foram alocadas no Grupo Treinamento foram submetidas a um protocolo de treinamento de força que teve a duração de dez semanas. Nas primeiras cinco semanas, a frequência semanal foi de duas vezes. Nas últimas cinco semanas, a frequência foi aumentada para três vezes semanais. Todas as sessões ocorreram em dias não consecutivos. O programa de treinamento foi planejado com base nas recomendações do *American Heart Association* ([WILLIAMS et al., 2007](#_ENREF_17)).

Previamente, ao início de cada sessão de treino, foi realizada uma rotina de aquecimento, caracterizada por uma caminhada, com duração de cinco minutos, numa intensidade correspondente a 60% da frequência cardíaca de reserva.

As técnicas dos exercícios foram ensinadas às participantes durante duas sessões de treino, separadas por 48 horas, realizadas, previamente, ao início do programa de treinamento. Essas sessões tiveram o objetivo de proporcionar às participantes uma familiarização com os exercícios. Sendo assim, em cada exercício foi realizada uma série de dez repetições, com a menor carga possível de cada aparelho, ensinando as idosas a adotarem um adequado posicionamento corporal, amplitude de movimento (considerando as limitações individuais), além de um correto padrão respiratório, durante a realização dos exercícios (evitando a manobra de Valsalva, inspirando na fase de relaxamento e expirando na fase de contração) ([COLADO; GARCIA-MASSO, 2009](#_ENREF_3)). Adicionalmente, essas sessões também tiveram a finalidade de ensinar a adequada interpretação da escala de percepção subjetiva de esforço (*OMNI-RES*), adaptada para treinamento de força ([ROBERTSON et al., 2003](#_ENREF_13)), a fim de que as idosas pudessem ter a intensidade do treinamento apropriadamente monitorada. Para facilitar o entendimento dessa escala subjetiva, foi aplicado o teste de 1RM no exercício Supino sentado e Leg press sentado, conforme protocolo adotado por [KRAEMER; FLECK (2009)](#_ENREF_7). Tal procedimento também serviu para verificar a eficiência do treinamento em relação aos ganhos de força das idosas.

Ao iniciar o programa de treinamento, as idosas alocadas no respectivo grupo, executaram nove exercícios pelo método de múltiplas séries os quais foram realizados seguindo a respectiva ordem de execução: Leg press sentado, remada sentada, flexão tronco, flexão joelho, supino sentado, extensão tronco, desenvolvimento de ombro, flexão plantar e puxada frente.

Em cada exercício, foi estabelecida, semanalmente, uma determinada faixa de repetições na qual as idosas realizaram as respectivas séries, numa intensidade submáxima, correspondente ao nível “moderado – um pouco difícil”, aferido pela escala de percepção subjetiva de esforço (*OMNI-RES*) ([ROBERTSON et al., 2003](#_ENREF_13)).

Quando a idosa alcançava confortavelmente o "limite superior" do intervalo de repetições prescrito, a carga de treinamento era aumentada por volta de 5%, conforme recomendação do *American Heart Association* ([WILLIAMS et al., 2007](#_ENREF_17)). O planejamento das variáveis intervenientes, no protocolo de treinamento de força, desta pesquisa, pode ser visualizado na Tabela 1.

| **Tabela 1** – Periodização do treinamento de força que foi realizado com as idosas hipertensas. | | | | | |
| --- | --- | --- | --- | --- | --- |
| Semana | Frequência semanal | Série | Repetição | Intervalo de descanso | PSE* |
| 1 | 2 | 1 | 9 – 11 | 120 segundos | 5 – 7 |
| 2 | 2 | 1 | 9 – 11 | 120 segundos | 5 – 7 |
| 3 | 2 | 2 | 9 – 11 | 120 segundos | 5 – 7 |
| 4 | 2 | 2 | 9 – 11 | 120 segundos | 5 – 7 |
| 5 | 2 | 2 | 11 – 13 | 90 segundos | 5 – 7 |
| 6 | 3 | 2 | 11 – 13 | 90 segundos | 5 – 7 |
| 7 | 3 | 2 | 11 – 13 | 90 segundos | 5 – 7 |
| 8 | 3 | 3 | 11 – 13 | 90 segundos | 5 – 7 |
| 9 | 3 | 3 | 13 – 15 | 60 segundos | 5 – 7 |
| 10 | 3 | 3 | 13 – 15 | 60 segundos | 5 – 7 |
| PSE* - Percepção subjetiva de esforço na escala *OMNI-RES* adaptada para treinamento de força. | | | | | |

**Análise estatística**

Os dados foram digitados, originalmente, no banco de dados do software SPSS^®^ (*Statistical Package for Social Sciences*) versão 21.0 para Windows. Após a estruturação final do banco de dados, foi realizada, inicialmente, uma análise descritiva de todos os dados relativos às variáveis dependentes e independentes. A verificação da normalidade dos dados foi aferida pelo teste de Shapiro-Wilk e por critérios adicionais de normalidade, adotados por [MARTÍNEZ-GONZÁLEZ; SÁNCHEZ-VILLEGAS; FAULÍN (2006)](#_ENREF_10).

As variáveis que apresentaram distribuição normal foram avaliadas pelo teste Split-Plot ANOVA (SPANOVA) “*mixed design*” o qual se trata de um modelo estatístico generalizado misto que considera um fator independente (grupo – treinamento vs controle) e o outro fator dependente ou correlacionado (tempo – pré vs pós). Caso fossem encontradas interações (grupo*tempo) significativas, seriam aplicados testes *post hoc*, entre os pares de comparação, através do Teste t de *Student* para amostras dependentes (intragrupo) e pelo Teste t de *Student* para amostras independentes (intergrupo). Quando na análise em questão, estiveram envolvidas comparações múltiplas, os valores de *p* encontrados foram submetidos à correção de *Bonferroni*. Naquelas variáveis que não apresentaram distribuição normal, a avaliação foi conduzida pelo Teste de Wilcoxon (intragrupo), e pelo Teste U de Mann-Whitney (intergrupo).

Nas variáveis FS e CV, foi calculada uma medida estatística denominada *effect size* (ES), no intuito de avaliar a magnitude da diferença entre os grupos (treinamento vs controle), no período pós-intervenção. Tal medida foi calculada através da seguinte fórmula (diferença média entre os grupos / desvio padrão agrupado). A classificação do ES foi determinada da seguinte maneira: 0,2 (baixo); 0,5 (moderado); 0,8 (alto); 1,3 (muito alto) ([SULLIVAN; FEINN, 2012](#_ENREF_15)).

A correlação entre as alterações no FS (ΔFS) e na CV basal (ΔCV) com as alterações nas variáveis sanguíneas (ΔPCRu, ΔNO_2_^-^, ΔMDA e ΔCAT) foram analisadas, através do coeficiente de correlação de Pearson (r), quando ambas as alterações apresentaram distribuição normal. Caso contrário, foi aplicado o coeficiente de correlação de Spearman (rho). Tais alterações foram consideradas no Grupo Treinamento, através da seguinte fórmula: (Δ = valor pós-treinamento – valor pré-treinamento). Para avaliar a força da correlação, entre as variáveis, foram considerados os seguintes valores: 0,2 (fraca); 0,5 (moderada); 0,8 (forte) ([SULLIVAN; FEINN, 2012](#_ENREF_15)).

Adicionalmente, a comparação de proporções, entre o uso de diferentes tipos de medicamentos anti-hipertensivos, consumidos pelas idosas, foi realizada pelo teste Exato de Fisher.

Em todas as situações, foi considerado um valor de significância menor do que 5%.

**REFERÊNCIAS**

BRAND-WILLIAMS, W.; CUVELIER, M.; BERSET, C. Use of a free radical method to evaluate antioxidant activity. **LWT-Food Science and Technology,** v. 28, n. 1, p. 25-30, 1995.

BREDT, D. S.; SNYDER, S. H. Nitric oxide: a physiologic messenger molecule. **Annu Rev Biochem,** v. 63, p. 175-95, 1994.

COLADO, J. C.; GARCIA-MASSO, X. Technique and safety aspects of resistance exercises: a systematic review of the literature. **Phys Sportsmed,** v. 37, n. 2, p. 104-11, Jun 2009.

COLDHAM, F.; LEWIS, J.; LEE, H. The reliability of one vs. three grip trials in symptomatic and asymptomatic subjects. **J Hand Ther,** v. 19, n. 3, p. 318-26; quiz 327, Jul-Sep 2006.

FRIEDEWALD, W. T.; LEVY, R. I.; FREDRICKSON, D. S. Estimation of the concentration of low-density lipoprotein cholesterol in plasma, without use of the preparative ultracentrifuge. **Clin Chem,** v. 18, n. 6, p. 499-502, Jun 1972.

JASPERSE, J. L. et al. Positional differences in reactive hyperemia provide insight into initial phase of exercise hyperemia. **J Appl Physiol (1985)**, p. jap.01253.2013, Jul 2 2015.

KRAEMER, W. J.; FLECK, S. J. **Otimizando o treinamento de força: programas de periodização não linear**. Manole, 2009. ISBN 8520427375.

LIMA, A. O. et al. **Métodos de laboratório aplicados à clínica: técnica e interpretaçäo**. Guanabara Koogan, 1992. ISBN 8527702258.

LUZ, P. L. D.; LAURINDO, F. R. M.; CHAGAS, A. C. P. Endotélio e doenças cardiovasculares. In: (Ed.). **Endotélio e doenças cardiovasculares**: Atheneu, 2003.

MARTÍNEZ-GONZÁLEZ, M. Á.; SÁNCHEZ-VILLEGAS, A.; FAULÍN, F. J. **Bioestadística amigable**. Díaz de Santos Madrid, 2006. ISBN 8479787910.

NEGRÃO, C. E. et al. Muscle metaboreflex control is diminished in normotensive obese women. **Am J Physiol Heart Circ Physiol,** v. 281, n. 2, p. H469-75, Aug 2001.

OHKAWA, H.; OHISHI, N.; YAGI, K. Assay for lipid peroxides in animal tissues by thiobarbituric acid reaction. **Anal Biochem,** v. 95, n. 2, p. 351-8, Jun 1979.

ROBERTSON, R. J. et al. Concurrent validation of the OMNI perceived exertion scale for resistance exercise. **Med Sci Sports Exerc,** v. 35, n. 2, p. 333-41, Feb 2003.

RONDON, M. U. et al. Abnormal muscle metaboreflex control of sympathetic activity in never-treated hypertensive subjects. **Am J Hypertens,** v. 19, n. 9, p. 951-7, Sep 2006.

SULLIVAN, G. M.; FEINN, R. Using Effect Size-or Why the P Value Is Not Enough. **J Grad Med Educ,** v. 4, n. 3, p. 279-82, Sep 2012.

TROMBETTA, I. C. et al. Weight loss improves neurovascular and muscle metaboreflex control in obesity. **Am J Physiol Heart Circ Physiol,** v. 285, n. 3, p. H974-82, Sep 2003.

WILLIAMS, M. A. et al. Resistance exercise in individuals with and without cardiovascular disease: 2007 update: a scientific statement from the American Heart Association Council on Clinical Cardiology and Council on Nutrition, Physical Activity, and Metabolism. **Circulation,** v. 116, n. 5, p. 572-84, Jul 31 2007.

ZITKUS, B. S. Update on the American Diabetes Association Standards of Medical Care. **Nurse Pract,** v. 39, n. 8, p. 22-32; quiz 32-3, Aug 16 2014.
