# Supplementary material for: Effect of Strength Training on Oxidative Stress and the Correlation of the Same with Forearm Vasodilatation and Blood Pressure of Hypertensive Elderly Women: A Randomized Clinical Trial
Source: PLoS One. 2016 Aug 16;11(8):e0161178. doi: 10.1371/journal.pone.0161178 (PMC4986983; doi:10.1371/journal.pone.0161178)
Supplement: S1 Table — PES—Perceived Exertion Scale (OMNI-RES) adapted for strength training. (DOC) [file pone.0161178.s005.doc]

**Supporting information 1**

| **Table 1.** **Linear reverse periodization of strength training that was held with elderly hypertensive women.** | | | | | |
| --- | --- | --- | --- | --- | --- |
| Week | Weekly Frequency | Sets | Repetition | Rest between sets | PES* |
| 1 | 2 | 1 | 9 – 11 | 120 seconds | 5 – 7 |
| 2 | 2 | 1 | 9 – 11 | 120 seconds | 5 – 7 |
| 3 | 2 | 2 | 9 – 11 | 120 seconds | 5 – 7 |
| 4 | 2 | 2 | 9 – 11 | 120 seconds | 5 – 7 |
| 5 | 2 | 2 | 11 – 13 | 90 seconds | 5 – 7 |
| 6 | 3 | 2 | 11 – 13 | 90 seconds | 5 – 7 |
| 7 | 3 | 2 | 11 – 13 | 90 seconds | 5 – 7 |
| 8 | 3 | 3 | 11 – 13 | 90 seconds | 5 – 7 |
| 9 | 3 | 3 | 13 – 15 | 60 seconds | 5 – 7 |
| 10 | 3 | 3 | 13 – 15 | 60 seconds | 5 – 7 |
| PES* - Perceived Exertion Scale (*OMNI-RES)* adapted for strength training. | | | | | |
